# Nuclear and mitochondrial tRNA-lookalikes in the human genome

**DOI:** 10.3389/fgene.2014.00344

**Published:** 2014-10-08

**Authors:** Aristeidis G. Telonis, Phillipe Loher, Yohei Kirino, Isidore Rigoutsos

**Affiliations:** Computational Medicine Center, Sidney Kimmel Medical College, Thomas Jefferson UniversityPhiladelphia, PA, USA

**Keywords:** tRNA, tRNA fragment, human genome, nuclear tRNA, mitochondrial tRNA

## Abstract

We are interested in identifying and characterizing loci of the human genome that harbor sequences resembling known mitochondrial and nuclear tRNAs. To this end, we used the known nuclear and mitochondrial tRNA genes (the “tRNA-Reference” set) to search for “*tRNA-lookalikes*” and found many such loci at different levels of sequence conservation. We find that the large majority of these tRNA-lookalikes resemble mitochondrial tRNAs and exhibit a skewed over-representation in favor of some mitochondrial anticodons. Our analysis shows that the tRNA-lookalikes have infiltrated specific chromosomes and are preferentially located in close proximity to known nuclear tRNAs (z-score ≤ −2.54, *P-value* ≤ 0.00394). Examination of the transcriptional potential of these tRNA-lookalike loci using public transcript annotations revealed that more than 20% of the lookalikes are transcribed as part of either known protein-coding pre-mRNAs, known lncRNAs, or known non-protein-coding RNAs, while public RNA-seq data perfectly agreed with the endpoints of tRNA-lookalikes. Interestingly, we found that tRNA-lookalikes are significantly *depleted* in known genetic variations associated with human health and disease whereas the known tRNAs are *enriched* in such variations. Lastly, a manual comparative analysis of the cloverleaf structure of several of the transcribed tRNA-lookalikes revealed no disruptive mutations suggesting the possibility that these loci give rise to functioning tRNA molecules.

## Introduction

The non-coding RNAs (ncRNAs) known as transfer RNAs (tRNAs) play critical roles in the translation of messenger RNAs (mRNAs) to amino acid sequences. Evolutionarily speaking, tRNAs are ancient molecules present in all three kingdoms of life (archaea, bacteria, eukaryota). Beyond their roles in protein synthesis, tRNAs have been found to also possess additional regulatory functions (Mei et al., 2010; Phizicky and Hopper, 2010; Durdevic and Schaefer, 2013; Raina and Ibba, 2014). More recently, fragments derived from tRNAs were also shown to regulate cellular physiology via pathways that are not yet fully understood (Gebetsberger and Polacek, 2013) or to be unexpectedly involved in pathways involved in the post-transcriptional regulation of transcript abundance (Maute et al., 2013). In terms of chromosomal organization, human tRNA genes tend to form clusters that contain multiple anticodon families whereas in terms of location they favor chromosomes 1 and 6 (Craig et al., 1989; Mungall et al., 2003).

From the standpoint of mutations, mitochondrial tRNA genes have long been implicated and/or directly linked with diseases with specific anticodons associated with the pathogenesis and molecular characteristics of syndromes and pathological conditions (Abbott et al., 2014). Even though mutations in nuclear tRNA genes have not been associated with any diseases (Abbott et al., 2014), human nuclear tRNAs have been shown to exhibit significant sequence diversity at the population level with functional consequences (Parisien et al., 2013).

The emerging complexity of tRNA biology suggests that it is important to delineate as accurately as possible the genomic “tRNA space” (i.e., the full complement of genomic locations harboring tRNA genes). Such ability will in turn facilitate the automated analyses of transcriptional next generation sequencing datasets. Traditionally, the emphasis of research efforts has been on a genome's *nuclear* tRNAs and tools such as the very successful tRNAscan-SE (Lowe and Eddy, 1997; Schattner et al., 2005; Chan and Lowe, 2009) greatly facilitated such analyses. Indeed, tRNAscan-SE can identify nuclear tRNA genes and tRNA pseudogenes across a very wide range of genomes and also predict the secondary structure of the respective transcripts. Currently, tRNAscan-SE is limited to identifying *nuclear* tRNAs and does not consider mitochondrial tRNAs whereas we wish to simultaneously search for lookalikes of the combined collection of *true* nuclear *and true* mitochondrial tRNAs.

The desire to examine the possibility of tRNA-lookalike loci in the nuclear genome by uniformly considering both the known *nuclear* and the known *mitochondrial* tRNA reference genes stems from the observation that both nuclear and mitochondrial tRNAs are biologically active entities, and that tRNA dynamics does not distinguish between the nucleus and the mitochondrion (Rubio and Hopper, 2011; Schneider, 2011). Mitochondrial tRNA-lookalikes in particular may be potentially significant due to the known role of mitochondrial tRNAs in diseases (Kirino et al., 2005; Abbott et al., 2014) but also because the concept of the mitochondrial-specific sequences/molecules may potentially be a source of erroneous biological conclusions due to the presence of the exact sequence/molecule in the nucleus (Yao et al., 2008).

There are many questions we seek to explore such as: Are some amino acids (anticodons) over-represented among the lookalikes, and, if so, which ones? Are any chromosomes “singled-out” in that they harbor a skewed number of nuclear/mitochondrial lookalikes compared to other chromosomes, and who are the responsible source tRNAs? Is there evidence of transcription for the loci harboring the lookalikes of nuclear/mitochondrial tRNAs? Lastly, are the lookalike loci enriched in known polymorphisms and/or disease linked mutations in analogy to what has been described for mitochondrial tRNAs?

## Materials and methods

### Search for genomic loci for mitochondrial and nuclear tRNA genes

We formed a “tRNA-Reference” set with 632 entries by combining the 22 known mitochondrial tRNA genes (NCBI entry NC_012920.1—http://www.ncbi.nlm.nih.gov/nuccore/251831106) with 610 of the 625 nuclear tRNAs from the GRCh37 (hg19) human genome assembly that are listed in gtRNAdb (Chan and Lowe, 2009). The 610 entries we sub-selected from gtRNAdb comprise 508 *true* tRNAs and 102 *pseudo*-tRNAs. The 25 entries of gtRNAdb that we excluded correspond to tRNAs with undetermined anticodon identity, tRNAs mapping to contigs that are not part of the major chromosome assembly, and the selenocysteine tRNAs. We stress that our inclusion of the 102 pseudo-tRNAs in the tRNA-Reference set is intentional: (a) it acknowledges the possibility that they may be functional (Rogers et al., 2012); (b) it helps avoid their “re-discovery” and simplifies the analysis and related bookkeeping; and, (c) it permits us to easily contrast in our analyses the attributes of lookalikes of true- and pseudo-tRNAs respectively (See Results). As mentioned above, the tRNAscan-SE package (Lowe and Eddy, 1997) currently does not handle mitochondrial tRNAs. In order to ensure consistency in the sensitivity of detection and in the thresholding we employed BLASTN (Altschul et al., 1990) using in turn each nuclear and mitochondrial sequence of the tRNA-Reference as a query. Such a search naturally gives rise to both partial- and full-length “hits” with varying lengths and levels of statistical significance. Of those hits we only kept the ones where the lookalike and the true tRNA query differed by not more than 2 nts in length: doing so enforces a near-similarity of lengths between a query and a lookalike and constrains any mismatches to the “*interior*” of the lookalike sequence. We define the number of mismatches as the length of the query sequence minus the number of identical bases between the query and the target sequence. Each tRNA-lookalike was associated with the most similar true tRNA from the tRNA-Reference collection.

### Overlaps with RepeatMasker

Any lookalikes that were identified by our search were compared with the 973 entries (full-length sequences and fragments) reported by RepeatMasker for the human genome GRCh37 (hg19) assembly (http://www.repeatmasker.org). Those tRNA-lookalikes that matched full-length RepeatMasker entries were identified and labeled as such.

### Density of tRNA-lookalikes across the nuclear chromosomes

For each chromosome we computed its “tRNA density” as the number of tRNA bases per million bases. Importantly, in this computation we *excluded* all pseudo-tRNAs and all pseudo-tRNA-lookalikes. Also, we counted separately the number of bases on the forward and reverse strands (= 2 ^*^ chromosome base-pairs). We computed this tRNA density in turn for each chromosome and for each number of allowed mismatches. The R package “gplots” was used to construct a heatmap for visualizing the results.

### Localization bias of tRNA-lookalikes across the nuclear chromosomes

First, we visualized on each nuclear chromosome the locations of all true tRNAs and all tRNA-lookalikes that our analysis uncovered. Each chromosome was depicted as a straight line along which we marked the location of each lookalike/true tRNA gene. To examine whether the tRNA-lookalikes have a tendency to localize in the vicinity of known tRNA genes, we performed the following Monte-Carlo simulation: we randomly chose 454 spots across the nuclear genome, computed for each spot its distance in basepairs to the closest true tRNA gene, and finally generated the average value of these distances. The process was repeated 1,000,000 times and allowed to numerically estimate the probability density function of the underlying distribution *D*. We also computed the average distance from true tRNAs for the tRNA-lookalikes and calculated its distance (z-score) from the mean value of the distribution *D*. Chromosomes 22 and Y were excluded from this simulation as they do not contain any true tRNA-Reference entries.

### Transcriptional characterization of the tRNA-lookalikes

We investigated the possibility of transcription by examining which of the tRNA-lookalike loci are part of known categories of transcripts. To this end, we considered the following categories: unspliced pre-mRNAs of protein-coding; non-protein-coding transcripts and long intergenic non-coding RNAs (lincRNAs) from Rel. 75 of ENSEMBL (Flicek et al., 2014); and, long non-coding RNAs (lncRNAs) from version 19 of GENCODE (Harrow et al., 2006). Those genomic regions that were present in more than one of these datasets were only considered once to avoid multiple-counting. We also examined the deep sequencing data that are available through the UCSC human genome browser (Bensasson et al., 2003) for evidence of expression based on the ENCODE RNA-seq track from the Cold Spring Harbor Laboratory (ENCODE Project Consortium, 2012). The data were obtained from whole cell as well as cytoplasmic RNA extracts from several distinct cell lines.

### Genomic variations in true tRNAs and tRNA-lookalikes

To investigate the enrichment of the tRNA-Reference and tRNA-lookalikes in single nucleotide polymorphisms (SNPs) and short variations we interested these two RNA datasets with build 141 of the NCBI dbSNP database (Sherry et al., 2001). To estimate the probability of the obtained fold enrichment values we numerically estimated the underlying probability density function using a Monte-Carlo simulation (10,000 iterations). We did this separately for each of the following four cases: (1) the true tRNAs from tRNA-Reference; (2) the pseudo-tRNAs from tRNA-Reference set; (3) the lookalikes of the true tRNAs; and, (4) the lookalikes of the pseudo-tRNAs We also repeated the analyses working with only the ClinVar database subset (Landrum et al., 2014) of dbSNP.

### Functional evaluation of some of the tRNA-lookalikes

A high-degree of similarity at the sequence level between a tRNA sequence and a tRNA-lookalike does not necessarily imply that the lookalike molecule, if transcribed, will function as a tRNA. Indeed, any mutations that may be present in the lookalike could lead to a loss of key attributes of the tRNA and thus have a deleterious effect on its ability to function as a tRNA. For example, the mutations could prevent proper folding of the transcript into a cloverleaf structure, disrupt Watson-Crick base pairing in the stems of the tRNA, adversely impact recognition of the enzymes involved in tRNA maturation such as aminoacyl-tRNA synthetase, etc. Manually investigating these possibilities is a demanding task that necessitates in-depth knowledge of the domain at hand. Moreover the required considerations increase very quickly with the number of allowed mismatches. Thus, to keep the task manageable, we examined only those of the lookalikes that had no more than five mismatches when compared to the most similar tRNA-Reference gene. In this evaluation, we distinguished among three possible categories. *Category A* comprised molecules that exhibited no change in their secondary structure, i.e., no unpaired bases where double helices are to be formed. *Category B* contained tRNA lookalikes with up to a single mismatch in the base pairing in the double helix that resulted in only mild changes to the cloverleaf's secondary-structure; these molecules *might* function, although not necessarily, as tRNAs as well. Lastly, *Category C* included tRNA-lookalikes containing mismatches that render these molecules highly unlikely to function as tRNAs even if they are transcribed. In all tRNA-lookalikes that we examined manually, there were no mutations in the anticodon triplet. If transcribed and cleaved appropriately, tRNA-lookalikes from Category A should have the highest potential to function as tRNA molecules.

## Results

### Many previously uncharacterized tRNA-lookalikes are present in the nuclear genome

**Figure 1:**
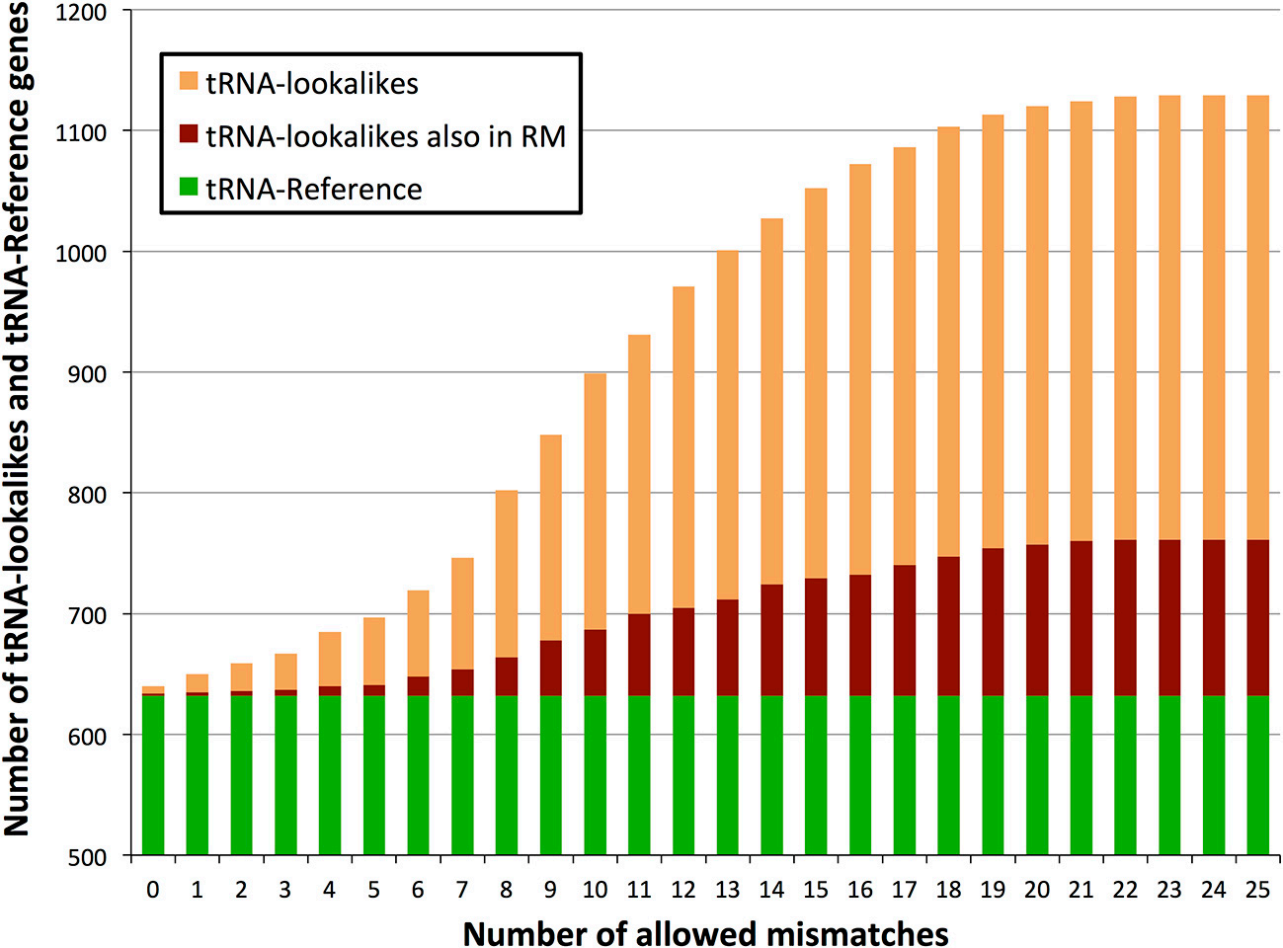
**Number of identified tRNA-lookalikes as a function of the allowed number of mismatches**. In addition to locating all 632 tRNA-Reference entries (green bars) we found many other nuclear genomic loci that harbored tRNA-lookalikes. Of the tRNA-lookalikes that we have identified, only a few (dark red bars) are currently labeled as tRNAs in RepeatMasker (RM) whereas the rest are novel. See Methods for the two filtering criteria (minimum length and maximum allowed mismatches) that we used.

We searched the human nuclear genome for lookalike instances of the entries in tRNA-Reference (See Methods), and, as expected, recovered all of its 632 entries. When allowing no mismatches, we found eight genomic loci that were identical (i.e., no mismatches) to a mitochondrial tRNA from the tRNA-Reference set. As we progressively relaxed the number of allowed mismatches we observed that initially the number of identified lookalikes increased but then reached a plateau around 23–25 mismatches (Figure 1); no loci could be identified with >25 mismatches. We found 497 tRNA-lookalikes with ≤25 mismatches: 454 of these corresponded to the 530 true tRNAs and 43 corresponded to the 102 pseudo-tRNAs contained in the tRNA-Reference set. Notably, of the 497 true-tRNA-lookalikes only 129 are listed among the RepeatMasker “tRNA” entries (See Methods and Figure 1) whereas the remaining 368 tRNA-lookalikes are novel. The entire table of the tRNA-Reference and tRNA-lookalike entries can be found in Supp. File S1.

### Non-uniform distribution of amino acids/anticodons among the tRNA-lookalikes

We associated each tRNA-lookalike with the tRNA-Reference entry with which it was most similar (See Methods). This allowed us to determine how the various tRNA-Reference entries were distributed across the human genome's real estate. In particular, we addressed the following two questions: (a) which tRNA-Reference entry can best serve as the “source template” of the tRNA-lookalike hit? (b) what is the genomic distribution of the tRNA-Reference and tRNA-lookalike sequences?

**Figure 2:**
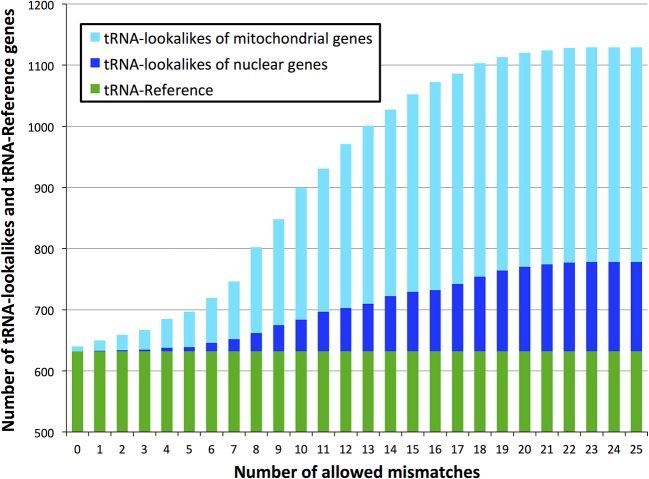
**Identity of the best matching source template**. Many of the discovered tRNA-lookalikes best resemble a nuclear tRNA source sequence (dark blue segments) with twice as many resembling a mitochondrial tRNA source (cyan segments).

With regard to the first question, despite there being more nuclear tRNAs than mitochondrial, we find that for many of the lookalikes of true tRNAs their source template is a mitochondrial tRNA from the tRNA-Reference set (Figure 2). Specifically, 351 tRNA-lookalikes best match one of the 22 mitochondrial tRNAs whereas 103 tRNA-lookalikes best match one of the 508 nuclear true tRNAs. A mere 43 lookalikes had their source template among the 102 pseudo-tRNAs in tRNA-Reference. It is worth noting here that mitochondrial tRNAs are ~67-fold over-represented among the tRNA-lookalikes compared to nuclear tRNAs. This enrichment was calculated as follows: (number of tRNA-lookalikes of mitochondrial origin/number of true mitochondrial tRNAs)/(number of tRNA-lookalikes of nuclear origin/number of true nuclear tRNAs).

**Figure 3:**
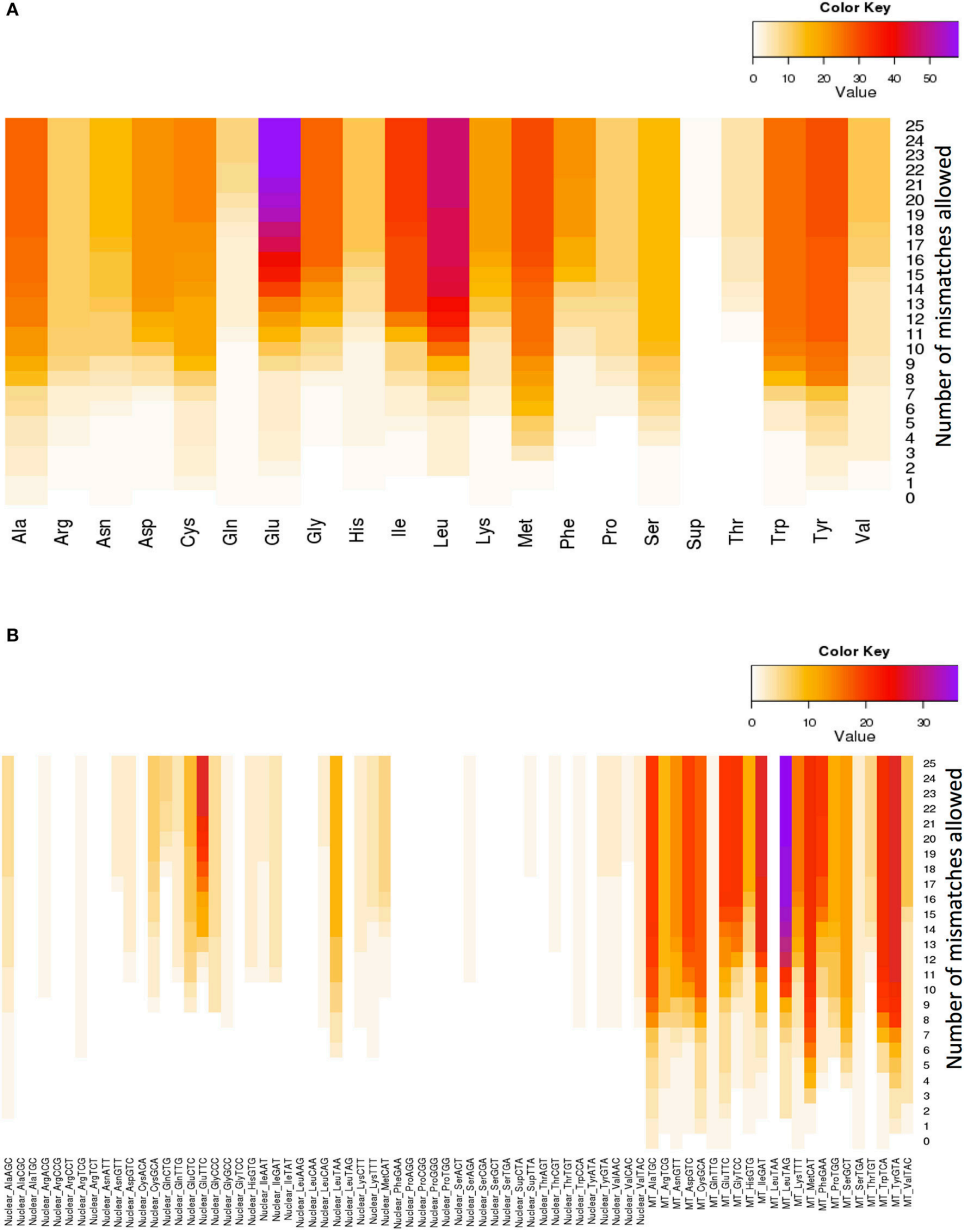
**Distribution of only the tRNA-lookalikes grouped by the coding amino acid**. **(A)** Distribution based on the coded amino acid. **(B)** Distribution based on the actual anticodon sequence that is used. In each panel the distribution is shown as a function of the allowed mismatches. No tRNA-Reference entries were included in this chart.

Glutamate (Glu) and leucine (Leu) are the amino acids whose tRNAs are the most represented among the lookalikes. The number of observed tRNA-lookalikes reaches a plateau between 10 and 15 mismatches (Figure 3A) with Glu being the exception. The observed plateau remains even when we group the tRNA-lookalikes by their anticodon (Figure 3B).

An interesting observation that can be made from Figure 3B is that distinct anticodons for the same amino acid generally have different numbers of tRNA lookalikes. One characteristic such example comes from the *nuclear* tRNA-lookalikes of Glu: the nuclear GluTTC has 27 instances among the tRNA-lookalikes whereas GluCTC has only 8 such instances. The situation is different for the *true* tRNAs of Glu: here, GluTTC and GluCTC are represented by the same number of entries in nuclear chromosomes, 13 each. The dominance of the mitochondrial tRNA genes in identifying lookalikes is also evident from Figure 3B.

In the mitochondrial genome, there are pairs of tRNA genes that are immediately adjacent to one another. What we found for such mitochondrial tRNA pairs is that they do not necessarily “migrate together” and the number of nuclear tRNA-lookalikes differs for each member of the pair. For example: the mitochondrial LeuTAG tRNA has 36 tRNA-lookalikes whereas its immediately adjacent mitochondrial neighbor, the SerGCT tRNA, has only 12 lookalikes. In this example the mitochondrial LeuTAG appears to be the more “rebellious” of the two because only 11 of its 36 nuclear lookalikes are immediately adjacent to and on the same strand as SerGCT's nuclear lookalikes, while nearly all SerGCT lookalikes (11 out of 12) are paired with a LeuTAG lookalike. Importantly, it is also worth noting that not all mitochondrial tRNAs have lookalikes. For example, for the criteria that we used in our analyses, the mitochondrial LeuTAA tRNA does not have any nuclear lookalikes.

The mitochondrial provenance of some of the discovered tRNA-lookalikes hearkens back to the previously reported nuclear mitochondrial DNA loci (NUMTs) and associated databases (Ramos et al., 2011). However, we point out that the genomic distribution of nuclear tRNA-lookalikes that we have uncovered generally follows that of the NUMTs. As an example, we note that 340 of the 351 nuclear lookalikes of mitochondrial tRNA that we uncovered are among the previously reported NUMTs (Ramos et al., 2011).

### Preferential penetration of specific chromosomes by tRNAs and tRNA-lookalikes

**Figure 4:**
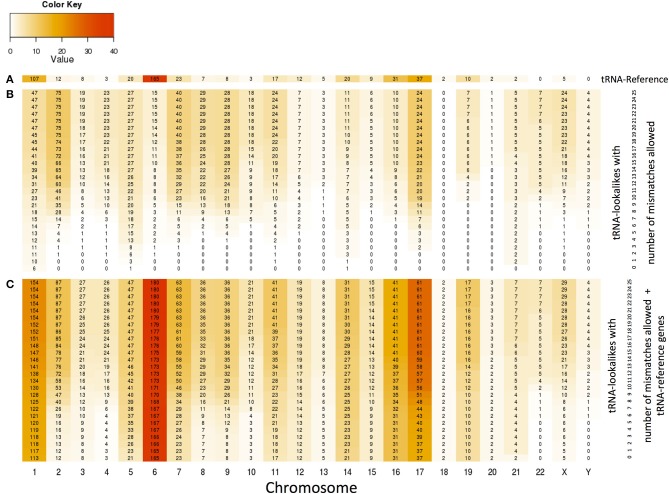
**Distribution and density of true tRNAs and lookalikes across the various chromosomes. (A)** Distribution of only the true tRNA-Reference entries across the chromosomes. **(B)** Distribution of only the tRNA-lookalikes that resemble true tRNAs across chromosomes shows the speed of chromosomal penetration by the lookalikes as a function of the allowed number of mismatches. **(C)** Distribution of the total number of tRNA instances (=true tRNA-Reference entries + tRNA-lookalikes) across the chromosomes. In all three panels, the number shown in each cell is the number of tRNA instances for the corresponding combination of chromosome and number of mismatches whereas the color of each cell represents the density of the chromosome in tRNA instances per million bases (See Methods).

With the collection of the 497 nuclear tRNA-lookalikes in hand, we determined, separately for each chromosome, the number of tRNA-Reference entries and also calculated the density of the tRNA Reference and tRNA-lookalike sequences per million bases. From this analysis, we excluded the pseudo-tRNA from tRNA-Reference as well as the nuclear lookalikes of pseudo-tRNAs. We find the presence of tRNAs and tRNA-lookalikes across chromosomes to be very uneven. For example, long chromosomes do not necessarily harbor the highest number of instances: chromosomes 1, 6, 7, 16, and 17 have the five top-most numbers of tRNA-Reference entries whereas chromosome 22 has none of them (Figure 4A and Supp. File S2A). Moreover, even though chromosome 1 is the longest of the human genome, it is chromosome 6 that has the highest *density* of tRNA-Reference entries, a result consistent with previous analyses of this chromosome's sequence (Mungall et al., 2003). When we consider how the tRNA-lookalikes penetrate the various chromosomes we find a somewhat complex situation (Figure 4B). Chromosomes 1, 2, 7, 8, and 9 have the five top-most numbers of tRNA-lookalikes whereas no lookalikes can be found in chromosome 18 even at the most tolerant setting of 25 allowed mismatches. We also found that as the number of allowed mismatches increases, some chromosomes (e.g., 2 and 7) exhibit a preferential enrichment in tRNA-lookalikes as compared to others (e.g., chromosomes 13 and 20). In Figure 4C we see the combined contribution of tRNA-Reference (true tRNAs only) and tRNA-lookalikes (of true tRNAs) to the total number of instances and corresponding chromosomal density across chromosomes: it is evident that chromosome 6, followed by chromosomes 1 and 17, contain the largest number of tRNA and tRNA-lookalike instances.

### Chromosomal co-localization of tRNA-lookalikes and tRNA-reference genes

We generated a genomic map showing the locations of true tRNAs and tRNA-lookalikes across the chromosomes (Supp. File S2A). As far as true tRNA genes are concerned, the known tRNA clusters on chromosomes 1 and 6 are clearly evident (Mungall et al., 2003). We also noticed additional clusters as well as what appeared to be a tendency of the tRNA-lookalikes to co-localize with true tRNA genes. Through a Monte Carlo simulation (See Methods) we evaluated the degree of genomic co-localization of tRNA-Reference and tRNA-lookalikes genomic loci. Supp. File S2B shows the resulting distribution of the average distance separating randomly selected spots from the closest true tRNA from the tRNA-Reference set—also shown (in red) is the actual average distance between the tRNA-lookalikes we have uncovered and true tRNAs; the latter is significantly smaller than what is obtained through randomly selected spots: z-score = −2.54 (*P*-value ≤ 0.00394). The finding indicates that the tRNA-lookalikes (a) are *not* randomly located in the human genome, and, (b) preferentially co-localize with true tRNAs from the tRNA-Reference set.

### Evidence of transcription for tRNA-lookalikes

**Figure 5:**
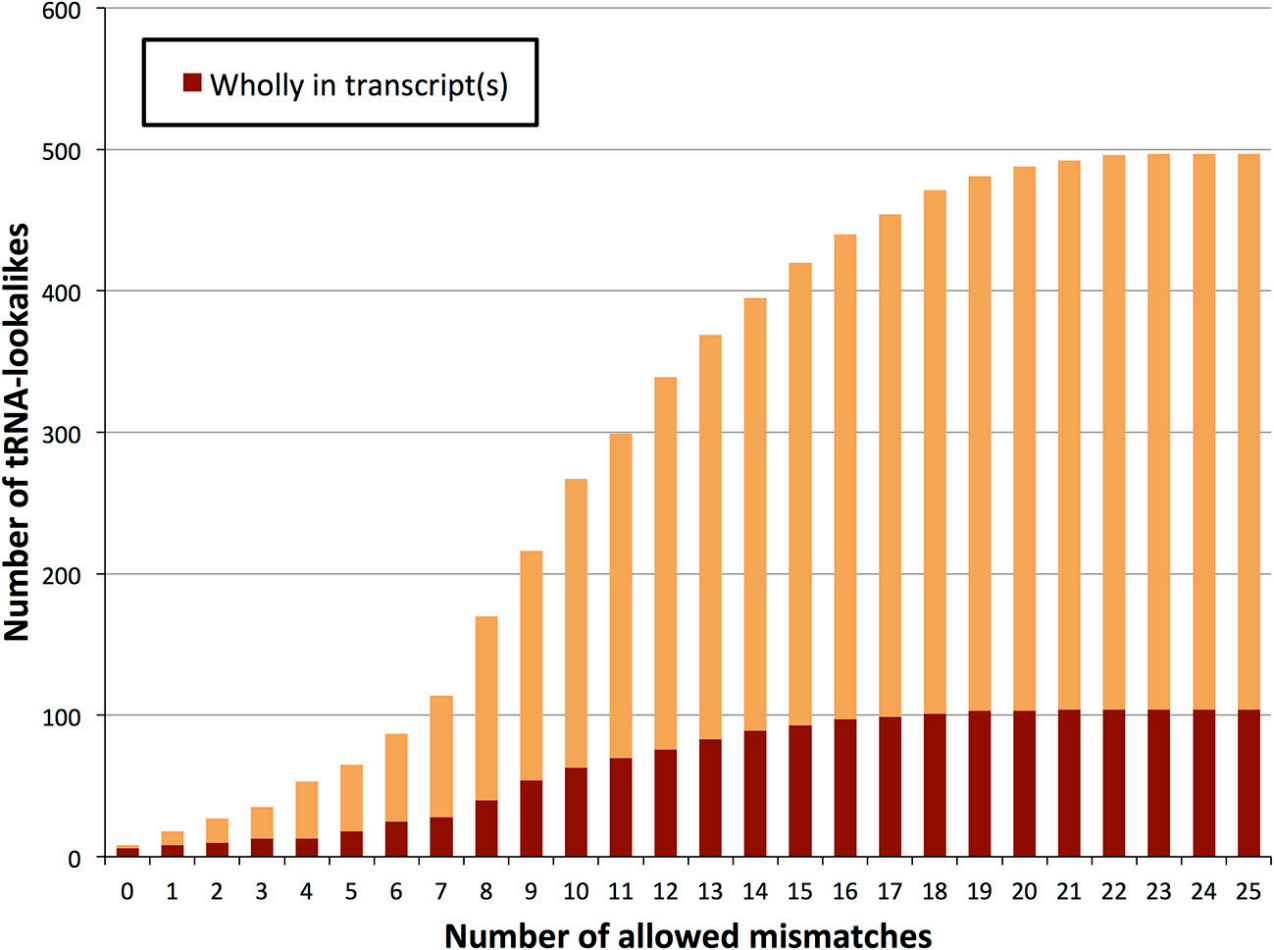
**TRNA-lookalikes and annotated RNA transcripts**. Distribution of the number of tRNA-lookalikes that are wholly present in known, annotated transcripts as a function of the number of allowed mismatches.

We also sought to determine which of the tRNA lookalikes are present in their entirety in known lincRNAs, lncRNAs, unspliced pri-mRNAs of protein coding transcripts, or other unspliced non-protein-coding transcripts. We find that more than 20% of the tRNA-lookalikes are part of at least one known, annotated transcript (Figure 5 and Supp. File S3). This percentage remains unchanged and independent of whether pseudo tRNAs are included in the computation or not. The unspliced pre-mRNAs of the JAK2 gene as well as the lincRNA RP5-857K21.4 include the highest number of tRNA-lookalikes (all these instances are due to lookalikes from true tRNAs). In all of the other cases, there were 4 or fewer lookalikes in the corresponding transcribed region of the non-coding RNA. We also performed the same analysis on the true tRNAs of the tRNA-Reference entries and found that 108 true tRNA-Reference genes are wholly contained in known transcripts. It is noteworthy that these 108 genes correspond to 20.4% of the true tRNA-Reference set, a percentage that is similar to what the tRNA-lookalikes that best matched to true tRNAs exhibited. Supp. File S3 also shows for each tRNA-Reference and tRNA-lookalike entry the corresponding identified annotated transcripts. Additional evidence of transcription was obtained through analysis of the ENCODE deep sequencing data generated by the Cold Spring Harbor Laboratory (ENCODE Project Consortium, 2012) that are available through the UCSC human genome browser. Manual searches readily revealed 26 additional examples where RNA-seq data perfectly matched the endpoints of our tRNA-lookalikes (Supp. File S4). In fact, 13 of these 26 examples are from the sequencing of *cytoplasmic* RNA extracts from five different cell lines suggesting the possibility that the corresponding loci are transcribed and likely processed similarly to *bona fide* tRNA molecules. We emphasize one important point. As evidenced by the same ENCODE RNA-seq data that we analyzed, not all true tRNAs are transcribed in all cell types: indeed, in Supp. File S5 we show several examples of tRNAs from the tRNA-Reference set that show no evidence of transcription in the cell lines that were deeply-sequenced by ENCODE. By the same token, one would not expect to see transcriptional evidence for all our tRNA-lookalikes either.

### Enrichment and depletion of SNPs in tRNAs and in tRNA-lookalikes

To further investigate the characteristics of the tRNA-lookalike loci, we examined whether the tRNA-Reference and tRNA-lookalikes were enriched in SNPs and small genetic variations. First, we considered the true tRNAs and the pseudo tRNAs from the tRNA-Reference set (Table 1). In both cases we found significant fold enrichment of 2.58 (*P*-value ≤ 10^−4^) and of 1.50 (*P*-value = 1.0^*^10^−4^) for the true tRNAs and the pseudo tRNAs respectively. When we tested the lookalikes, we did not find a significant fold enrichment in SNP for either for the lookalikes of true tRNAs or the lookalikes of pseudo tRNAs (Table 1).

**Table 1:** **Enrichment of tRNAs in entries of the dbSNP and its subset of ClinVar**.

	**Database**			
	**dbSNP**		**ClinVar**	
	**Fold enrichment**	***P*-value**	**Fold enrichment**	***P*-value**
True tRNAs	2.582	≤ 10^−4^	2.500	≤ 10^−4^
Pseudo tRNAs	1.502	1.0 × 10^−4^	1.273	4.0 × 10^−3^
Lookalikes from true tRNAs	1.083	1.3 × 10^−3^	0.719	4.9 × 10^−3^
Lookalikes from pseudo tRNAs	1.127	3.0 × 10^−4^	0.000	–

Due to the heterogeneity of the entries in dbSNP, we sub-selected the subset of variations that are also present in the ClinVar database (Landrum et al., 2014) and repeated our analysis. The true and pseudo tRNAs from the tRNA-Reference set exhibited the same trend as with the whole dbSNP (Table 1). However, the lookalikes of true tRNAs were found to show depleted (0.719 fold enrichment or 1.39-fold reduction, *P*-value = 4.9 × 10^−3^)—see Table 1. Interestingly, none of the more than 50 K entries in ClinVar overlapped with any of the lookalikes of a pseudo tRNA.

### Potential functionality of tRNA-lookalikes

**Figure 6:**
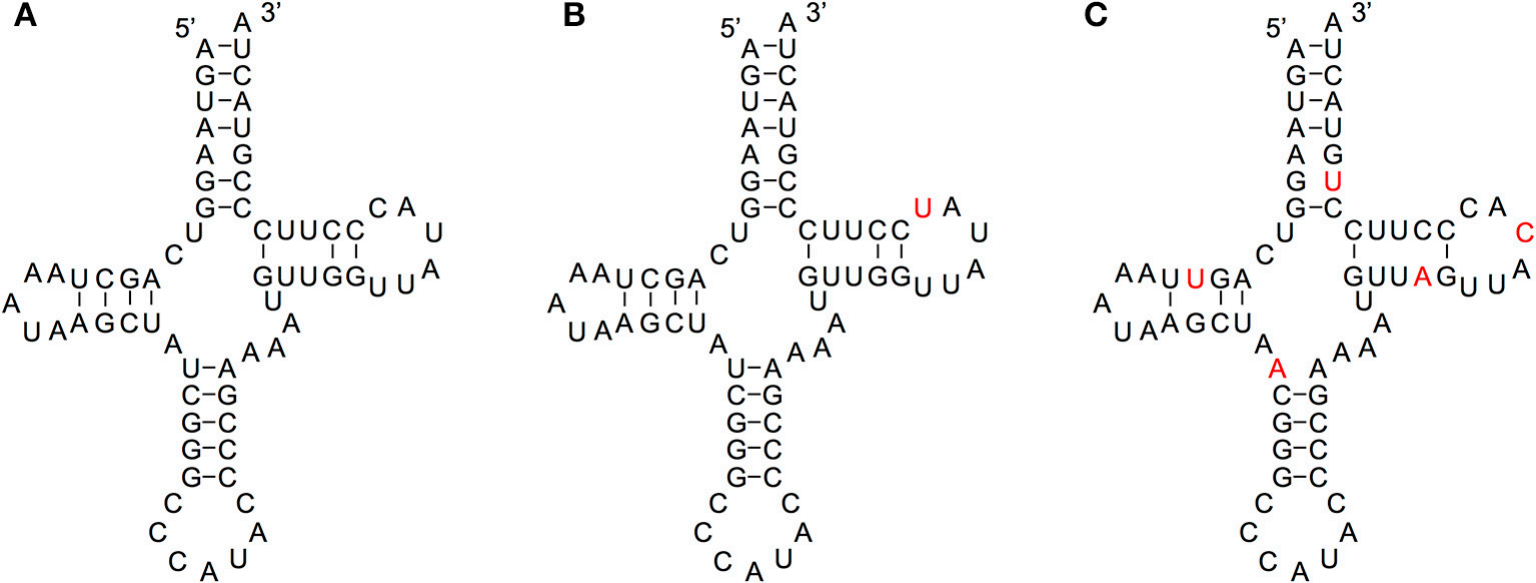
**Examining the functional potential of tRNA-lookalikes**. Examples of the mitochondrial tRNA-Reference entry for Methionine **(A)** and of two tRNA-lookalike hypothesized secondary structures shown in panels **(B,C)**. The nucleotides shown in red correspond to sequence differences from the reference gene. The lookalike in **(B)** belongs to Category A, while the one in **(C)** belongs to Category C (See Methods). The lookalike shown in panel **(B)** is located on the forward (+) strand of chromosome 1, between positions 564952 and 565019 inclusive; the lookalike shown in panel **(C)** is located on the reverse (–) strand of chromosome 9, between positions 81357661 and 81357728 inclusive.

We compared the secondary structures of tRNA-lookalikes to the secondary structure of the corresponding most similar entry from the tRNA-Reference set and manually evaluated the results. Given the complexity of the task and the non-automatable nature of this step, we considered only the 65 lookalikes of true tRNAs that had up to five mismatches compared to their counterpart best matching tRNA-Reference entry. This decision was dictated by the fact that in addition to requiring in-depth knowledge of the domain at hand, the complexity of the task increases very quickly with the number of allowed mismatches. Based on the results of our analysis, we were able to divide these 65 sequences among the three categories (A, B, and C—See Methods) as follows. In Category A we included lookalikes that exhibit no significant disruptions of the secondary-structure. In Category B we included lookalikes with evident yet moderate secondary structure changes. Lastly, in Category C we included lookalikes that due to a significant number of disrupting mutations are most likely *not* functional molecules. While additional experiments would be needed to determine if lookalikes function as tRNAs, the ones from Category A should have the highest potential to function as tRNA molecules. Of the 65 tRNA-lookalikes, 39 fall in groups A or B (Figure 6 and Supp. File S6), suggesting the possibility that functioning cloverleaf structures may arise from these loci. Notably, in all 65 tRNA-lookalikes, the anticodon tri-nucleotide matched exactly that of the best matching entry from the tRNA-Reference dataset. Out of the total 65 tRNA-lookalikes in this group, 18 are wholly contained in annotated RNA transcripts whereas 12 of the 18 belong to Category A or Category B, and are possibly functional. Finally, we note that 7 of the 18 transcribed tRNA-lookalikes are located on chromosome 1 and all 7 but 1 belong to Category A.

### On-line data availability

**Figure 7:**
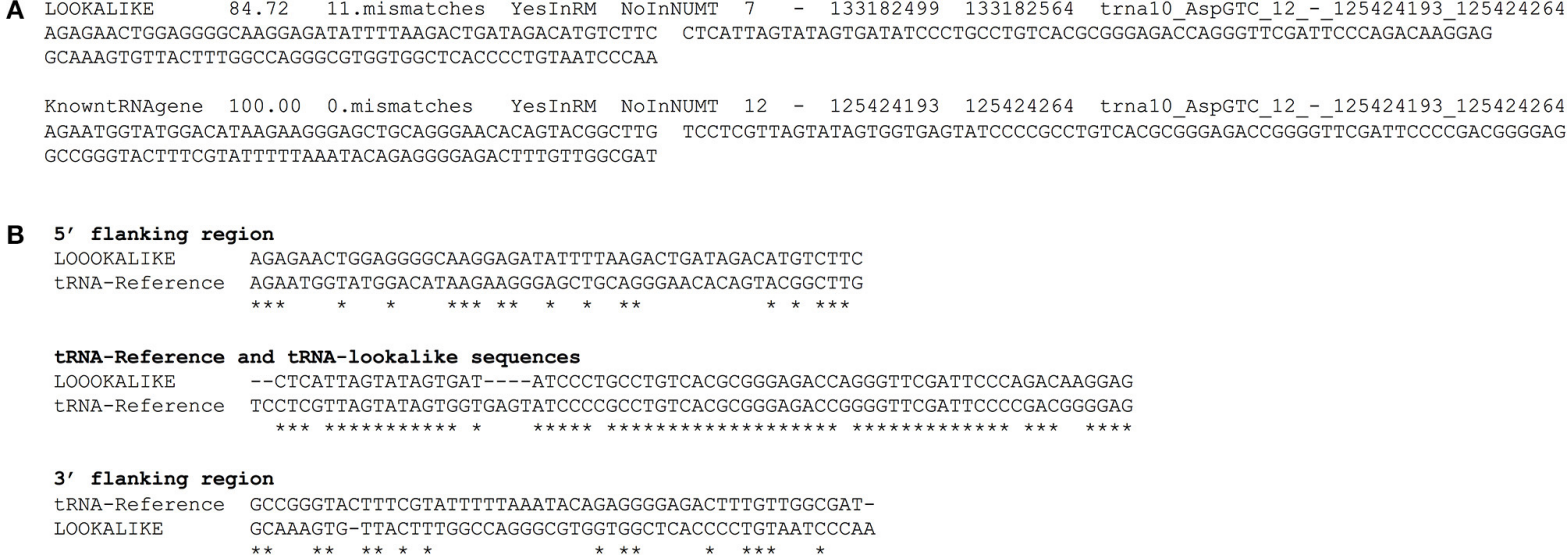
**Examples from the compiled dataset. (A)** Example of two entries, corresponding to a tRNA-lookalike and a tRNA-Reference gene (KnowntRNAgene) respectively from the database that we have generated. The entries correspond to an AspGTC tRNA. The shown columns, from left to right, contain a description of the genetic locus, the percentage identity and the number of mismatches (i.e., number of non-identical bases with the best tRNA-Reference entry) with the best-matched hit, the presence (or absence) in RepeatMasker (RM) or in NUMT, the genomic location (chromosome, strand, “from” and “to” coordinate, the best-matching hit (if a lookalike) or the name of the tRNA (if the entry is already in tRNA-Reference), the sequence of 50 nts up-stream of the hit and of 50 nts down-stream of it. **(B)** Clustal-W alignment for the tRNA and its lookalike and separately for each of its two flanking regions. Asterisks indicate identical bases and dashes indicate gaps. The anticodon of the shown tRNA is GTC and shown here boldfaced and underlined in context: […]ATCCCCGCCT**GTC**ACGCGGGAGACC[…]. All sequences are shown in 5′ to 3′ orientation.

We have compiled a table with the genomic coordinates for all the tRNA-lookalikes up to 25 mismatches. For each lookalike we report the sequence of the tRNA-lookalike and its genomic coordinates, a flanking region of 50 nts upstream, and a second flanking region of 50 nts downstream of it. We also report: the percentage identity between the lookalike and the best matching tRNA-Reference entry; the number of mismatches between the lookalike and the reference entry; and whether the lookalike is present in a known NUMT. Figure 7A shows an example of two lines from this database that relate to an AspGTC tRNA. Figure 7B shows a Clustal-W alignment for the tRNA (trna10-AspGTC) and its lookalike and separately for each of the two flanking regions. Note how the similarity of the tRNA-lookalike to trna10-AspGTC (central segment) does *not* extend to either the upstream or the downstream flanking region. The complete dataset comprising the above-mentioned information can be found in Supp. File S1 and is also available on-line in the form of a tab-delimited table and can be accessed from: http://cm.jefferson.edu/tRNA-lookalikes/.

## Discussion

In this study we sought to enhance our knowledge of the tRNA space by investigating the possibility that loci of the nuclear genome contain lookalikes of known nuclear and mitochondrial tRNAs. We identify 497 such distinct loci. Of these loci, 76% are not present among RepeatMasker's labeled entries.

Our analysis suggests that these tRNA-lookalikes are likely not random events. Indeed, we find that the various anticodons are not uniformly represented among the tRNA-lookalikes. As a matter of fact, specific anticodon groups have more tRNA-lookalikes than others. For example, when allowing up to 25 mismatches and without excluding lookalikes of pseudo tRNAs, SerTGA has 15 nuclear lookalikes whereas SerGCT has 12 and SerAGA has only one. An analogous trend is seen with e.g., Arg: ArgTCG has 10 lookalikes whereas each of ArgACG and ArgCCT has only one lookalike.

The collection of tRNA-lookalikes that we identified comprises lookalikes of both nuclear and mitochondrial tRNAs. However, the majority of the lookalikes best resemble mitochondrial tRNAs. Even so, it is worth noting that the observed genomic distributions and resulting chromosomal densities of the lookalikes that we uncovered follows those of the previously reported NUMTs (Bensasson et al., 2003; Parr et al., 2006; Ramos et al., 2011; Tsuji et al., 2012). The existence of mitochondrial tRNA-lookalikes in the nuclear genome is by itself an important subject considering that mutations in mitochondrial tRNAs have been linked to diseases (Belostotsky et al., 2012; Abbott et al., 2014). It remains to be seen whether such tRNA-lookalikes are transcribed and have functional roles.

The tRNA-lookalikes that we uncovered appears to favor specific chromosomes and their chromosomal distribution differs from that of the tRNA-Reference genes. We found chromosomes 2, 7, and 17 to be most dense in tRNA-lookalikes (Figure 4B). Chromosome 18 is special, in that it is the only chromosome with no tRNA-lookalikes in it. The tRNA-Reference genes on the other hand are most dense on chromosome 6 (Mungall et al., 2003) followed by chromosomes 1, 17, and 16. This skewed enrichment of chromosomes in true tRNAs and in tRNA-lookalikes might be rooted in the spatial organization of chromosomes in the nucleus, similar to what has been described in yeast (Chen and Gartenberg, 2014); or, it might be due to as-yet-unexplored roles of tRNA genes (Van Bortle and Corces, 2012). With regard to genomic localization, the known clusters of tRNA genes in chromosome 1 and 6 (Mungall et al., 2003) seem to attract tRNA-lookalikes (Supp. Files S1, S2). Nonetheless, there are also chromosomes with scattered tRNA-lookalikes (e.g., chromosome X). Notably, we find that the average distance of the tRNA-lookalikes to the closest true tRNA-Reference gene is significantly smaller than what is expected by chance suggesting that these tRNA-lookalikes are not random events and their genomic preferences may be driven by the same events that are behind the known clusters of true tRNAs (Mungall et al., 2003).

We also examined the possibility that the tRNA-lookalike loci may be transcribed. We find that approximately 20% of the lookalikes are wholly contained in the unspliced mRNA transcripts of important protein-coding genes, or in annotated non-coding RNAs, and thus these loci are transcribed. In particular, the JAK2 protein-coding gene and the RP5-857K21.4 lincRNA contain several tRNA-lookalikes each in their transcribed regions. To answer whether functional tRNA molecules are produced requires additional and lengthy experimental investigations. Nonetheless, we were able to identify intriguing evidence for 26 of our uncovered tRNA-lookalikes (none of which is currently in RepeatMasker) with the help of the ENCODE RNA-seq data. The evidence strongly supports the transcription and possible tRNA nature of these 26 tRNA-lookalikes especially since 13 of the deep-sequenced datasets were generated from cytoplasmic RNA extracts.

We also used the dbSNP database to examine whether tRNAs and tRNA-lookalikes are enriched in genetic variations. We found that the true tRNAs exhibited a significant enrichment about 1.5 times more than expected in a random genomic region (*P*-value ≤ 10^−4^). This result further supports the observed variation of human tRNAs at the population level (Parisien et al., 2013). However, the lookalikes of true tRNAs were depleted in them (1.39× – *P*-value = 4.9 × 10^−3^). Finally, lookalikes of pseudo tRNAs did not overlap with any of the ClinVar entries. Arguably, these findings suggest that the presence of genetic variations in tRNA-lookalikes warrants further research. Moreover, in light of the emerging role of classic pseudogenes (Pink and Carter, 2013) and of pseudo tRNAs (Rogers et al., 2012) in regulating cellular processes, and in conjunction with this study's findings, pseudo tRNAs ought to be considered in future analyses.

We also investigate whether these tRNA-lookalikes have the potential to be functional. To this end, we manually inspected the sequence alignments and the secondary structures of several dozen tRNA-lookalikes and identified 39 that can fold into proper cloverleaves. For 13 of these 43, publicly available data provides evidence that transcription occurs at their loci. Whether such molecules become part of the tRNA biogenesis pathway and its dynamics (Hopper et al., 2010), or whether they can be modified like true tRNAs (Jackman and Alfonzo, 2013) is the focus of future research in our laboratory.

Before embarking on tRNA biology studies from a genomics perspective, it is important that the genomic regions that share similarities with the known tRNAs be accurately defined. Our finding that some of the lookalike loci are transcribed suggests the importance of augmenting the publicly available databases and we have thus assembled all the information described in this paper and make it publicly available. Clearly, with regard to the possible tRNA-ness of these novel sequences, significant additional experimental effort will be required before it can be established beyond a doubt which of the loci that we have identified possess properties ascribed to tRNAs and are able to generate fully functional tRNA molecules. This is a topic of ongoing research activity in our laboratory.

### Conflict of interest statement

The authors declare that the research was conducted in the absence of any commercial or financial relationships that could be construed as a potential conflict of interest.
