# Supplementary material for: Nuclear and mitochondrial tRNA-lookalikes in the human genome
Source: Front Genet. 2014 Oct 8;5:344. doi: 10.3389/fgene.2014.00344 (PMC4189335; doi:10.3389/fgene.2014.00344)
Supplement: Supplementary file 2 [file DataSheet2.PDF]

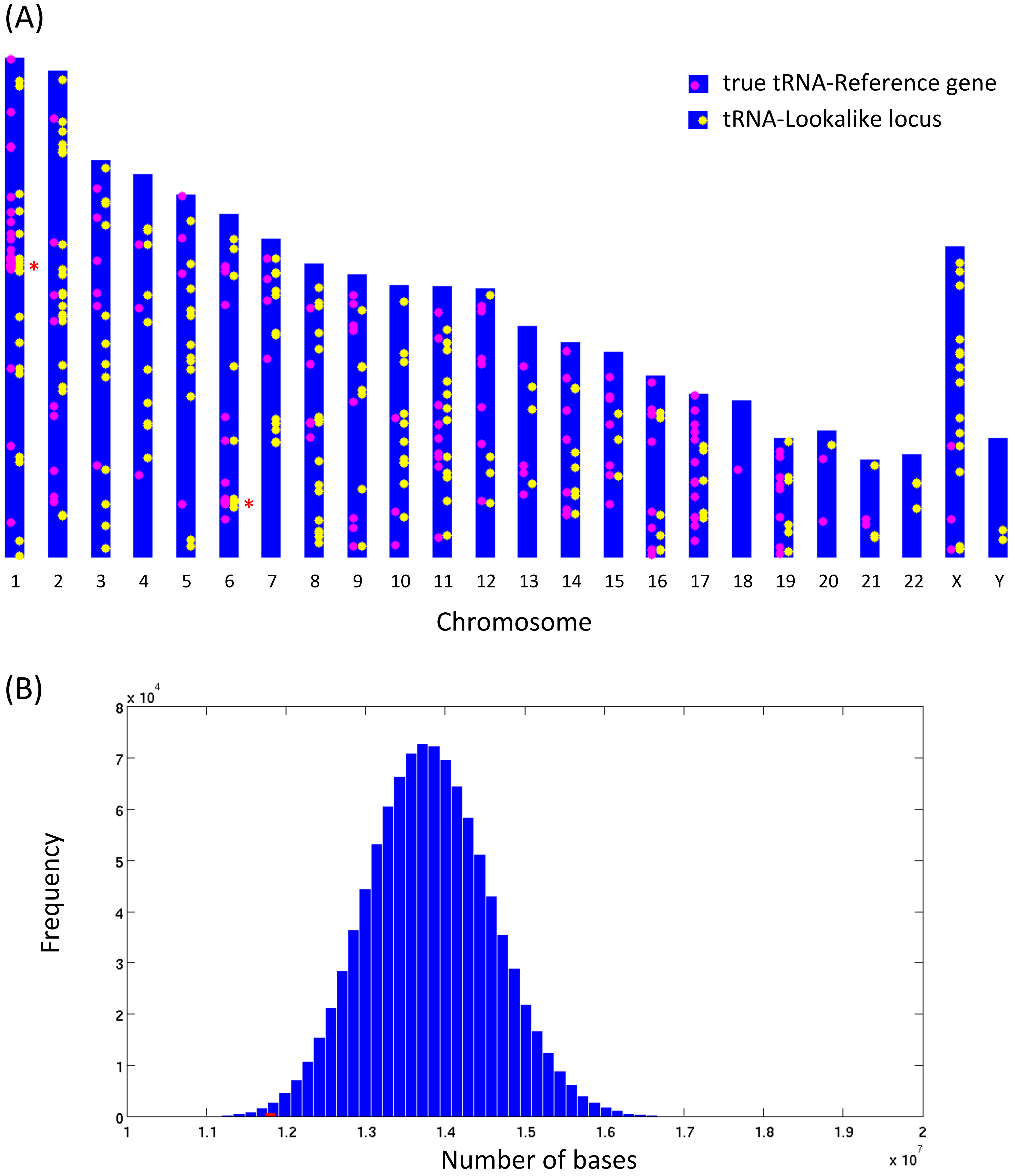

Supp. File S2: (A) The position of true tRNAs genes (purple) and their lookalikes (yellow) across nuclear chromosomes. For each chromosome, the position +1 is at the bottom. Stars (*) indicate examples of co-localization of true tRNA genes and tRNA-lookalikes. (B) The probability distribution (blue) of the average distance of 500 randomly selected spots to the closest tRNA-Reference gene. Mean of the distribution: 13804920 base pairs, standard deviation: 787172. The average distance of the tRNA-Lookalikes from the closest tRNA-Reference gene (red) is 11805663 base pairs. The Z-score of the tRNA-Lookalikes as to the distribution is 2.54
