# Supplementary material for: Nuclear and mitochondrial tRNA-lookalikes in the human genome
Source: Front Genet. 2014 Oct 8;5:344. doi: 10.3389/fgene.2014.00344 (PMC4189335; doi:10.3389/fgene.2014.00344)
Supplement: Supplementary file 4 [file DataSheet4.PDF]

Supp. File S4

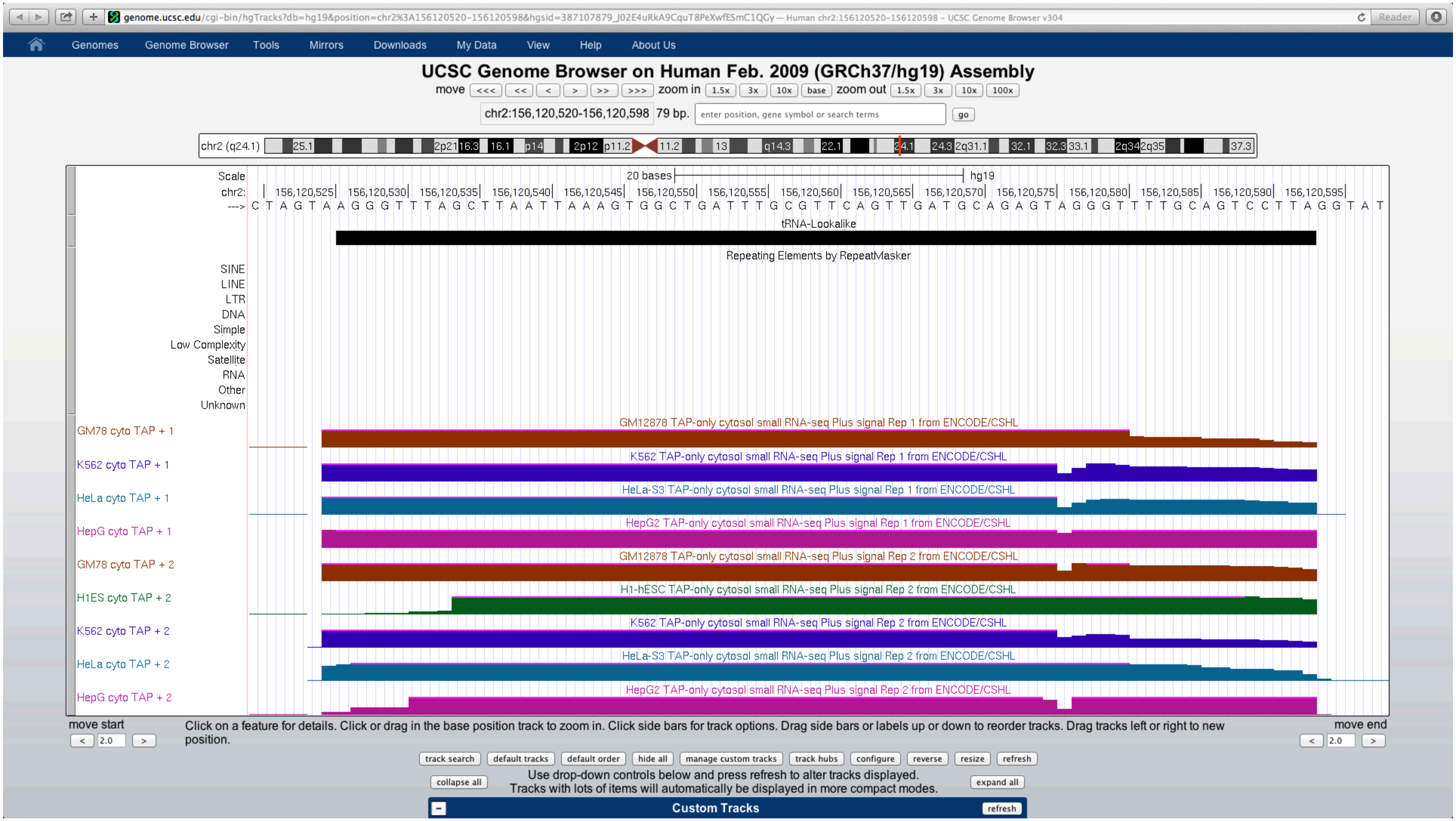

AlaTGC chrMT strand (-) 5587-5655 / tRNA-Lookalike at chr2, strand (+) 156120525-156120593

Supp. File S4

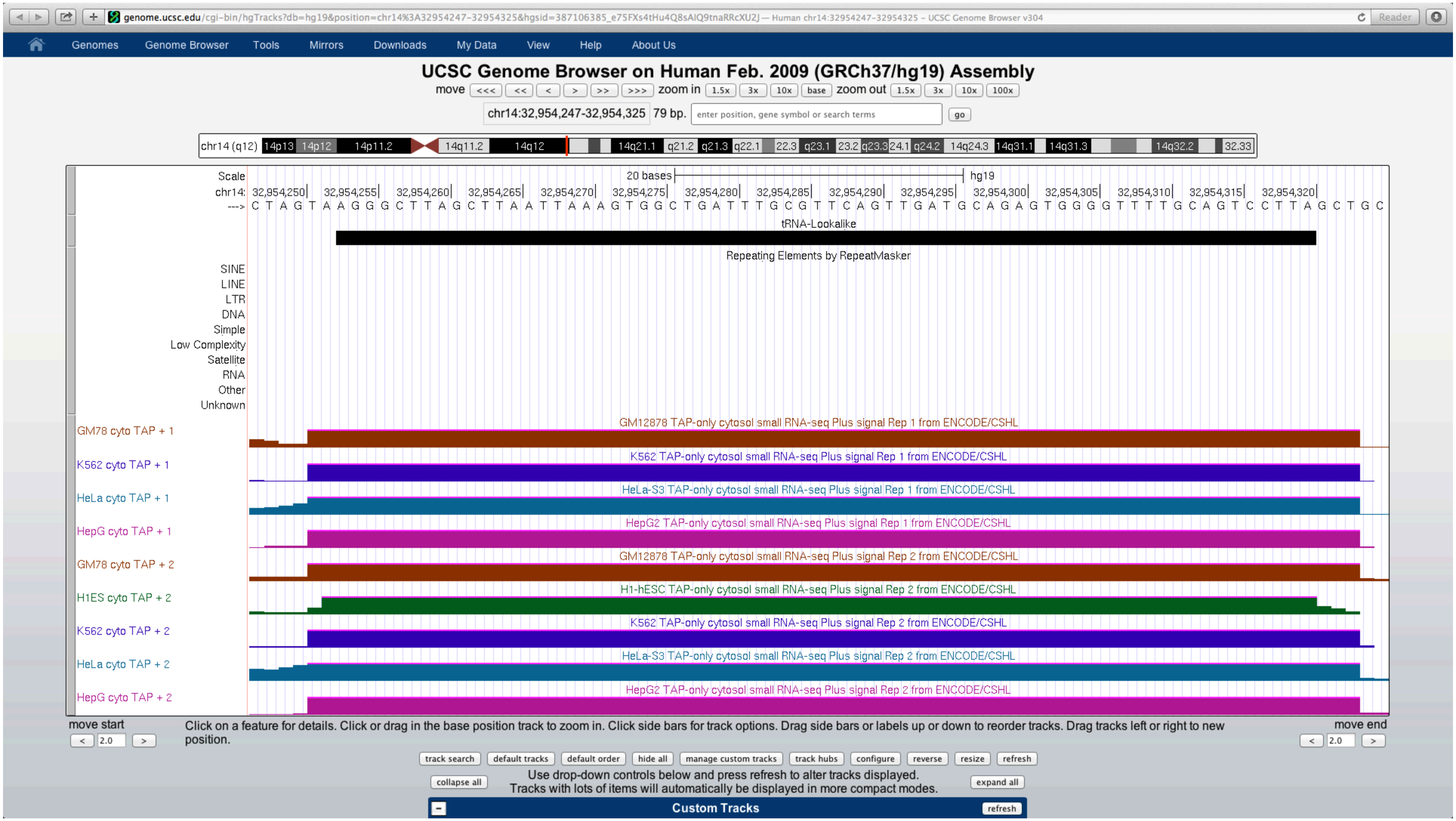

AlaTGC chrMT strand (-) 5587-5655 / tRNA-Lookalike at chr14, strand (+) 32954252-32954320

Supp. File S4

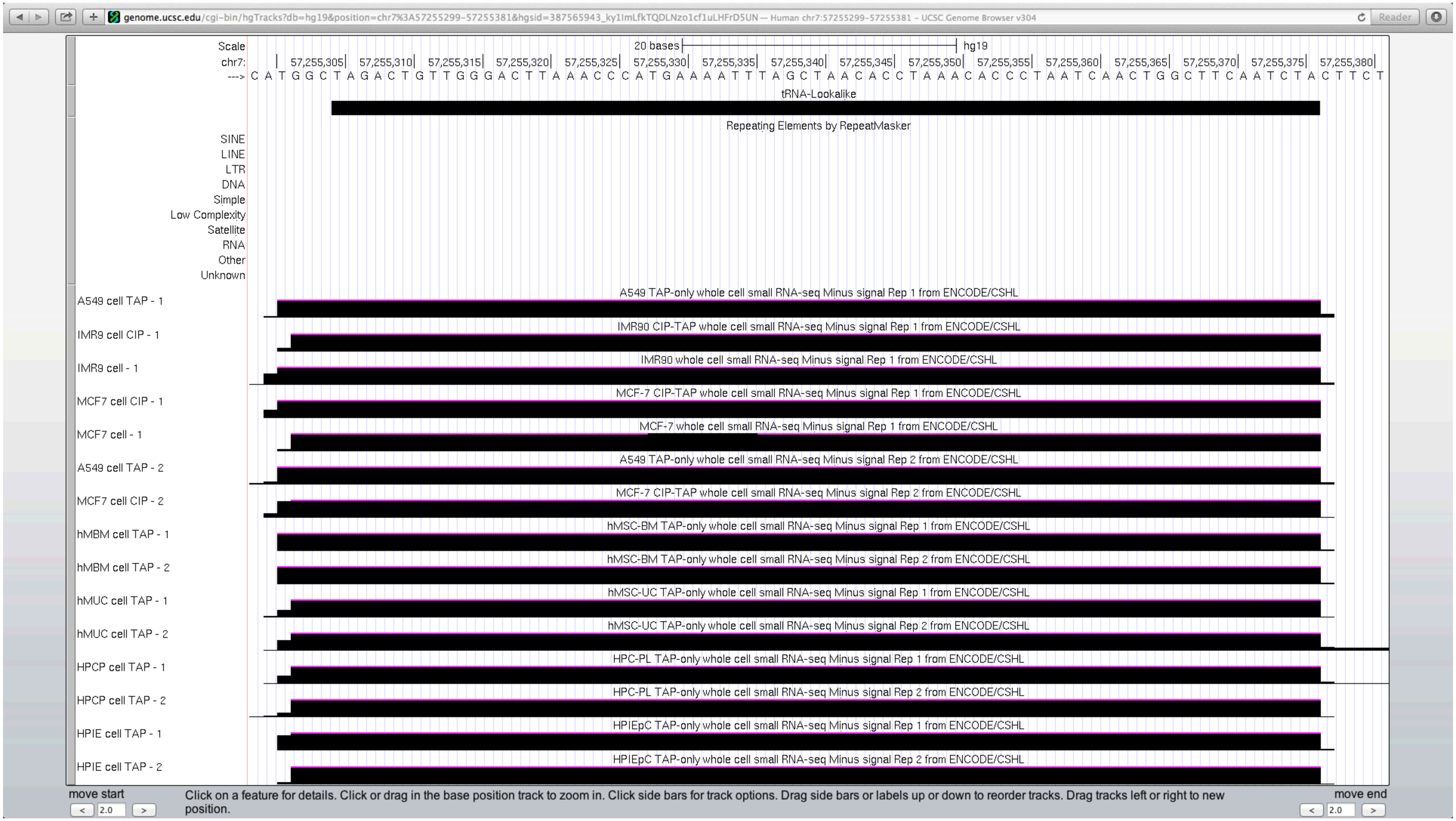

AsnGTT chrMT strand (-) 5657-5729 / tRNA-Lookalike at chr7, strand (-) 57255304-57255376

Supp. File S4

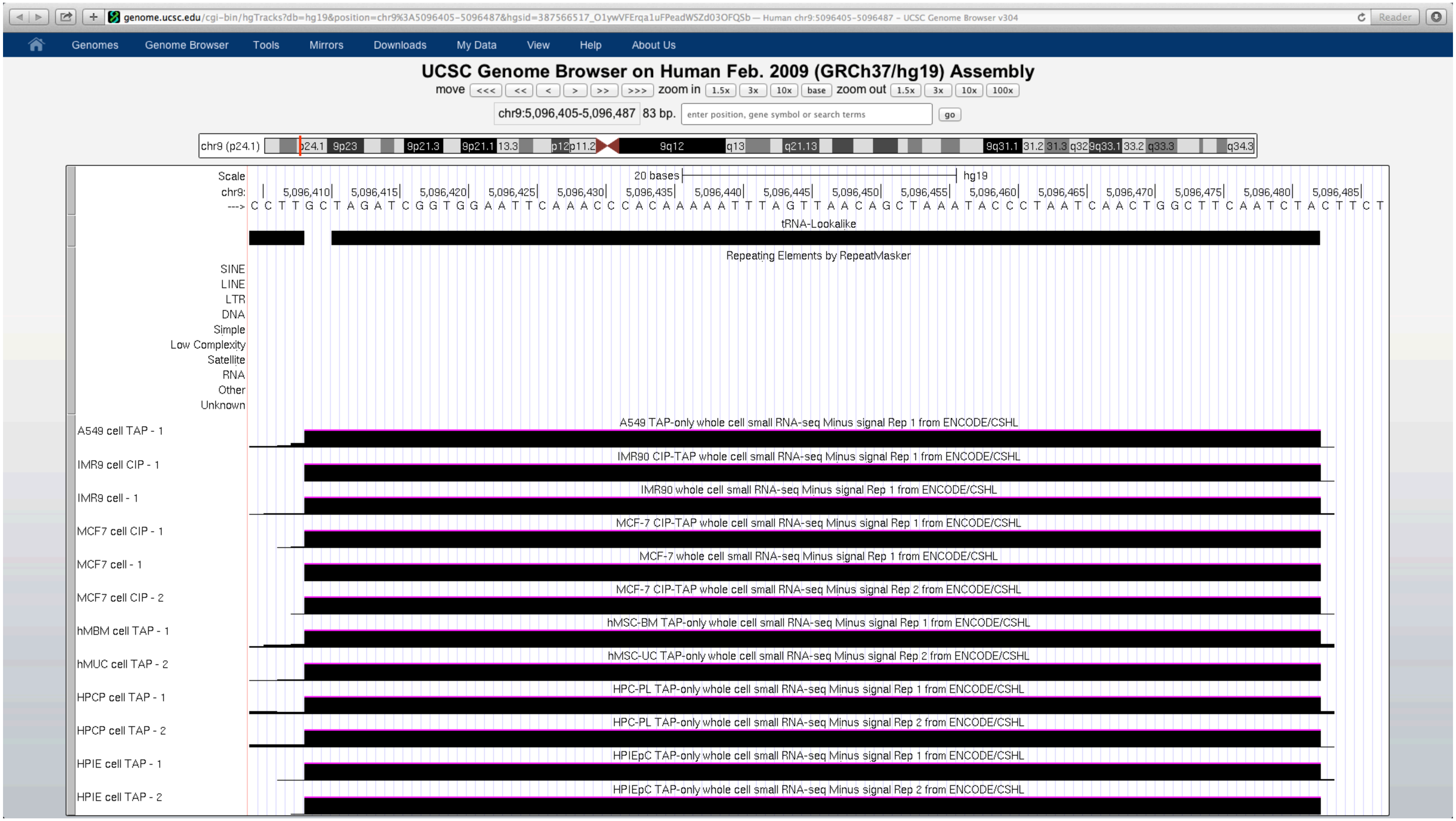

AsnGTT chrMT strand (-) 5657-5729 / tRNA-Lookalike at chr9, strand (-) 5096410-5096482

Supp. File S4

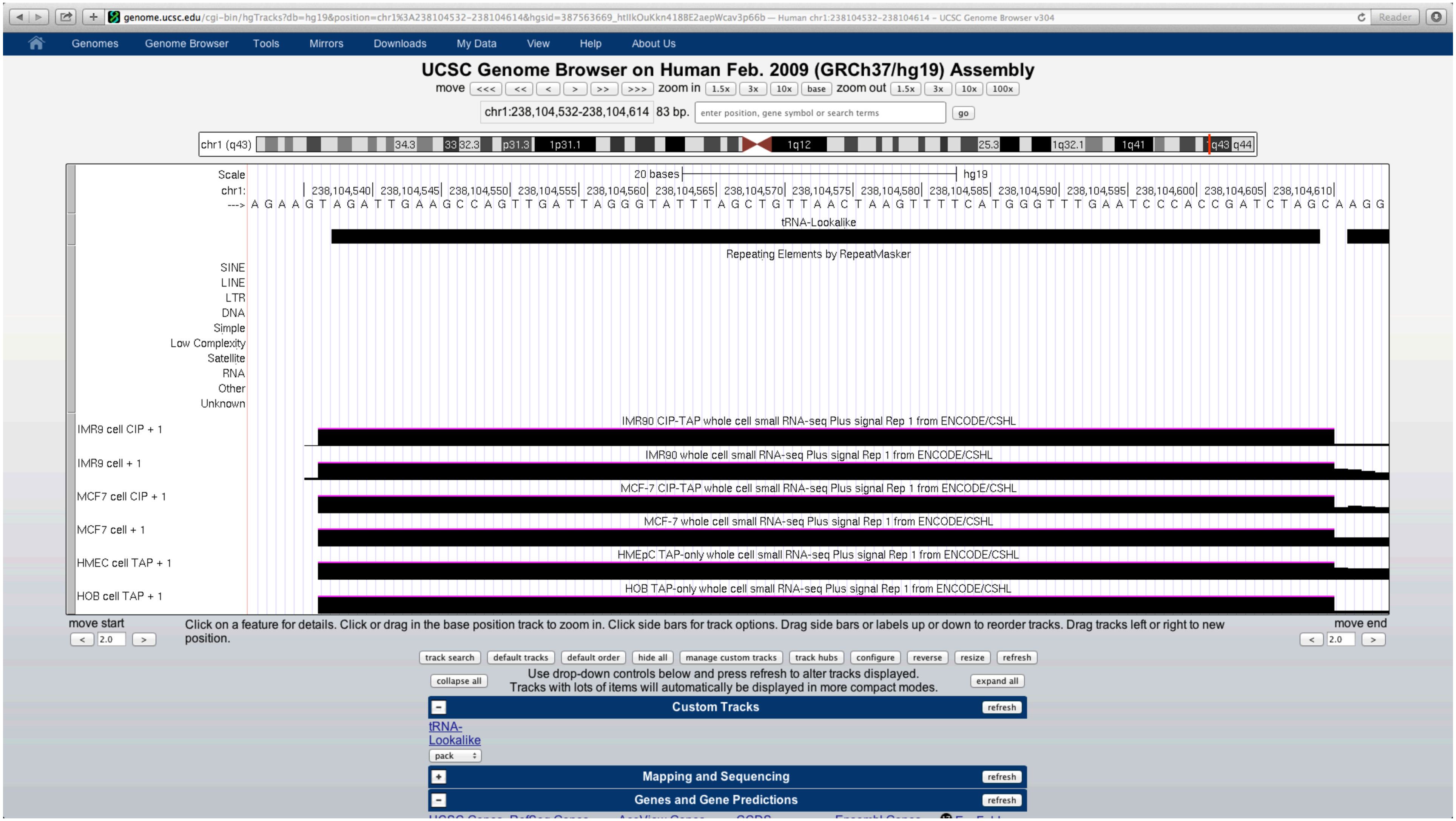

AsnGTT chrMT strand (-) 5657-5729 / tRNA-Lookalike at chr1, strand (+) 238104537-238104609

Supp. File S4

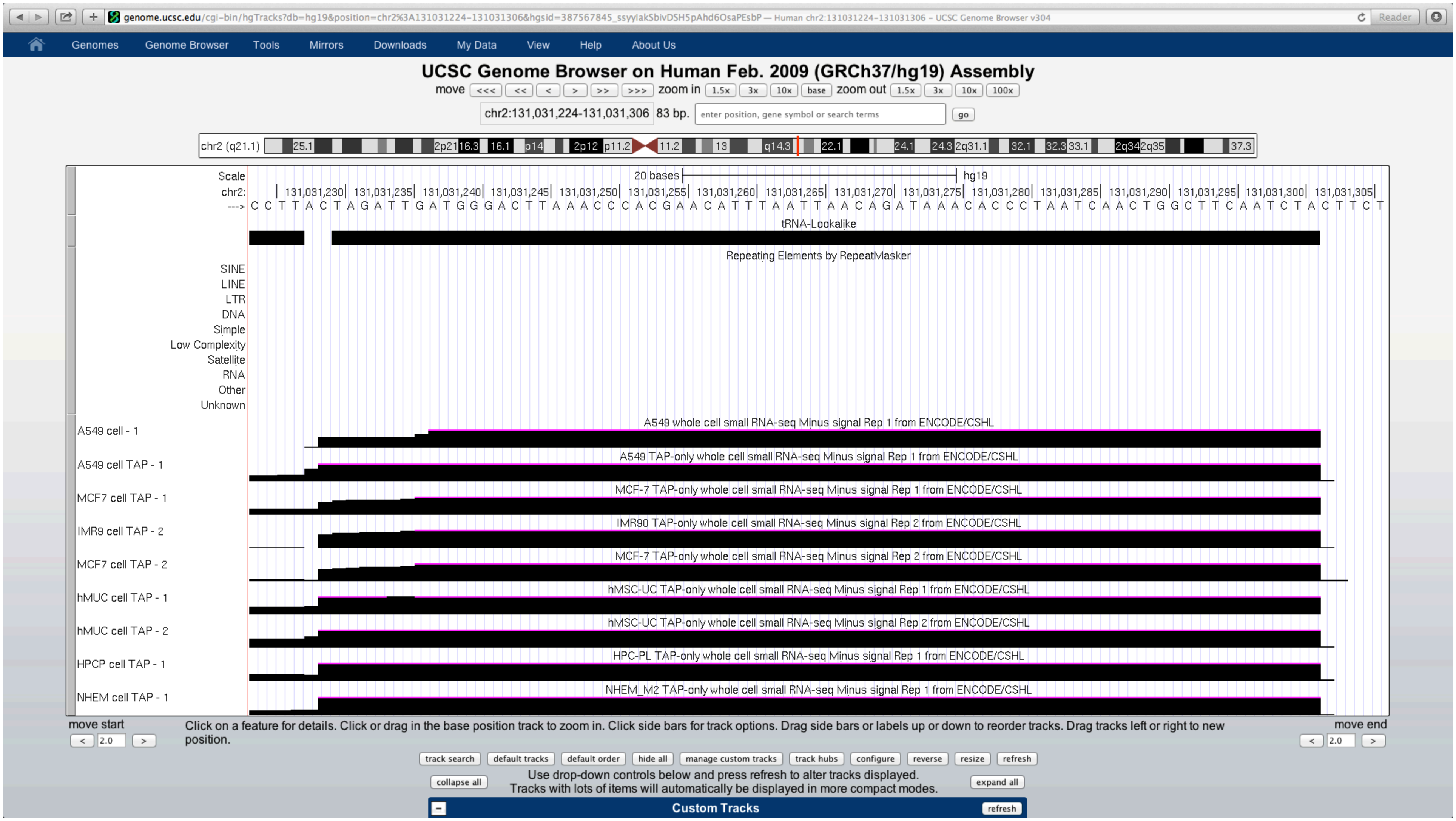

AsnGTT chrMT strand (-) 5657-5729 / tRNA-Lookalike at chr2, strand (-) 131031229-131031301

Supp. File S4

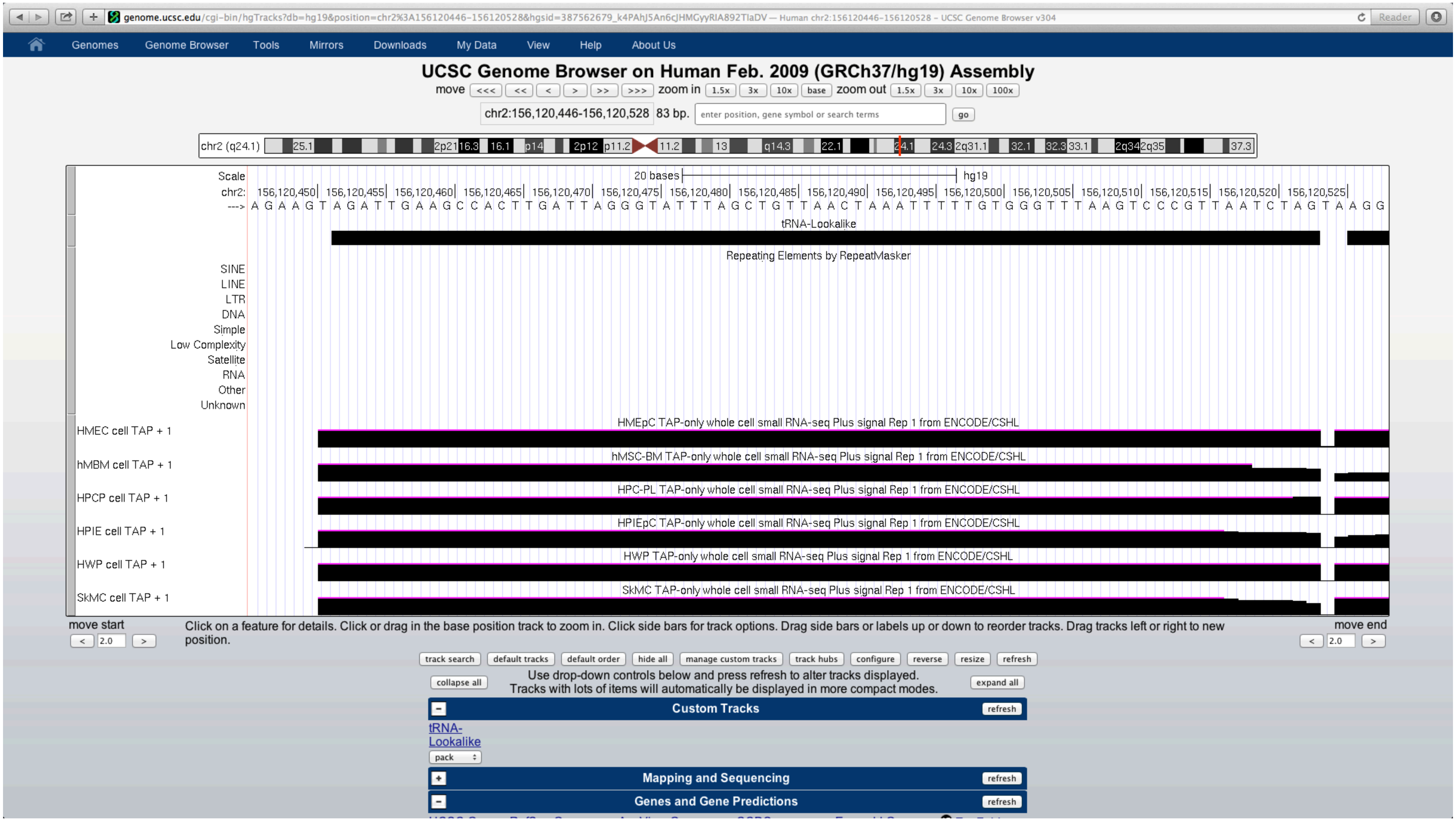

AsnGTT chrMT strand (-) 5657-5729 / tRNA-Lookalike at chr2, strand (+) 156120451-156120523

Supp. File S4

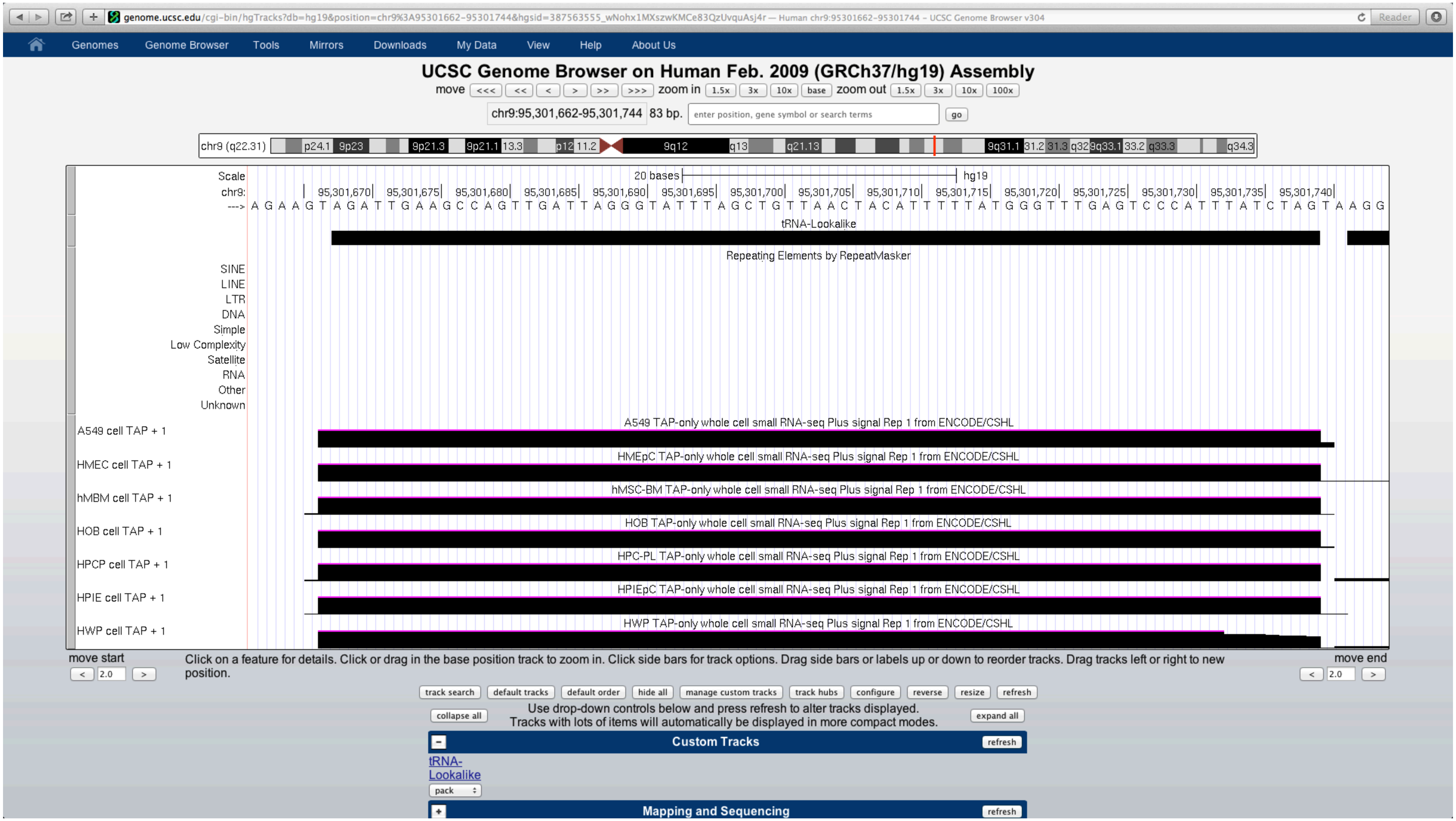

AsnGTT chrMT strand (-) 5657-5729 / tRNA-Lookalike at chr9, strand (+) 95301667-95301739

Supp. File S4

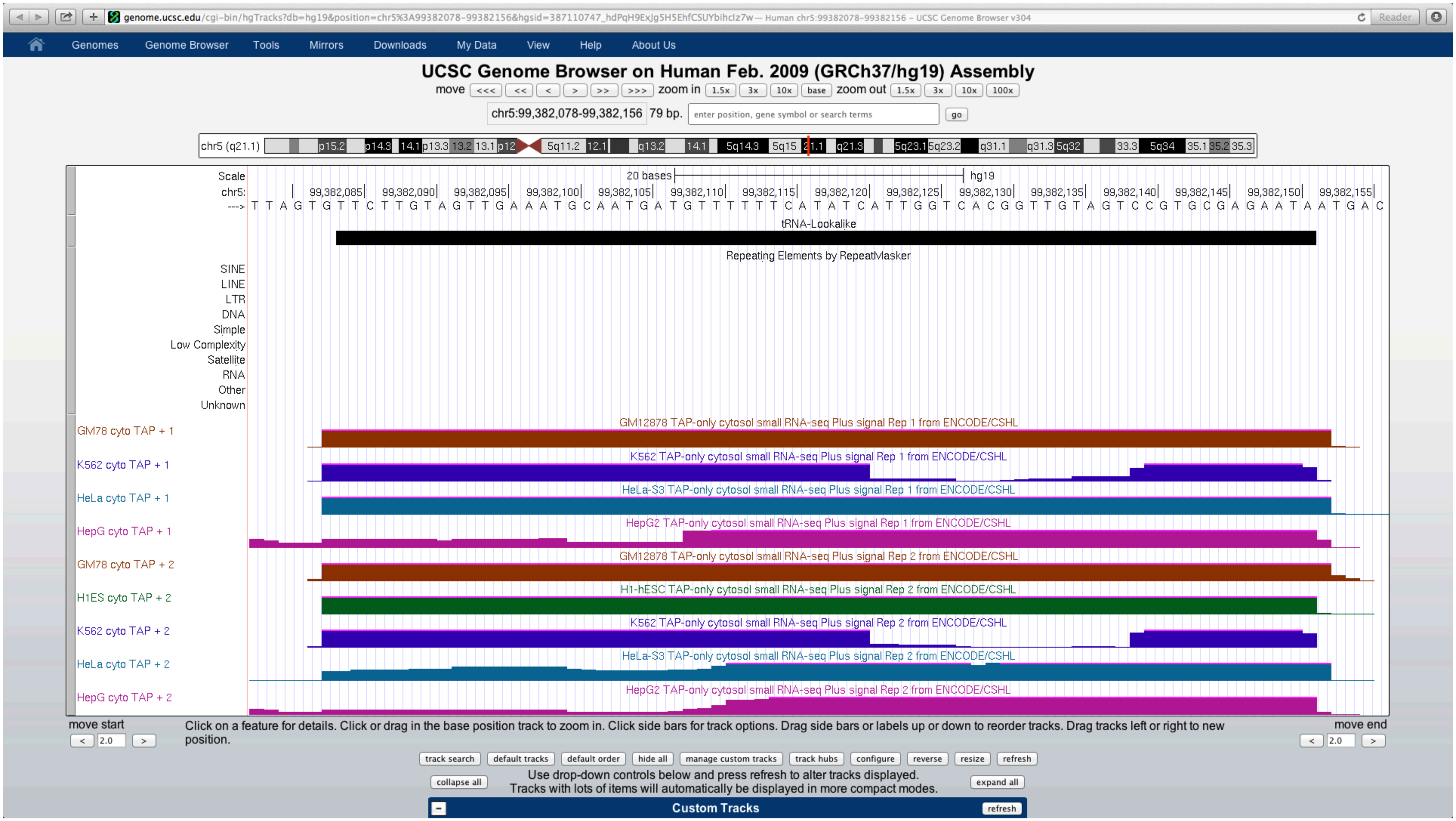

GluTTC chrMT strand (-) 14674-14742 / tRNA-Lookalike at chr5, strand (+) 99382083-99382151

Supp. File S4

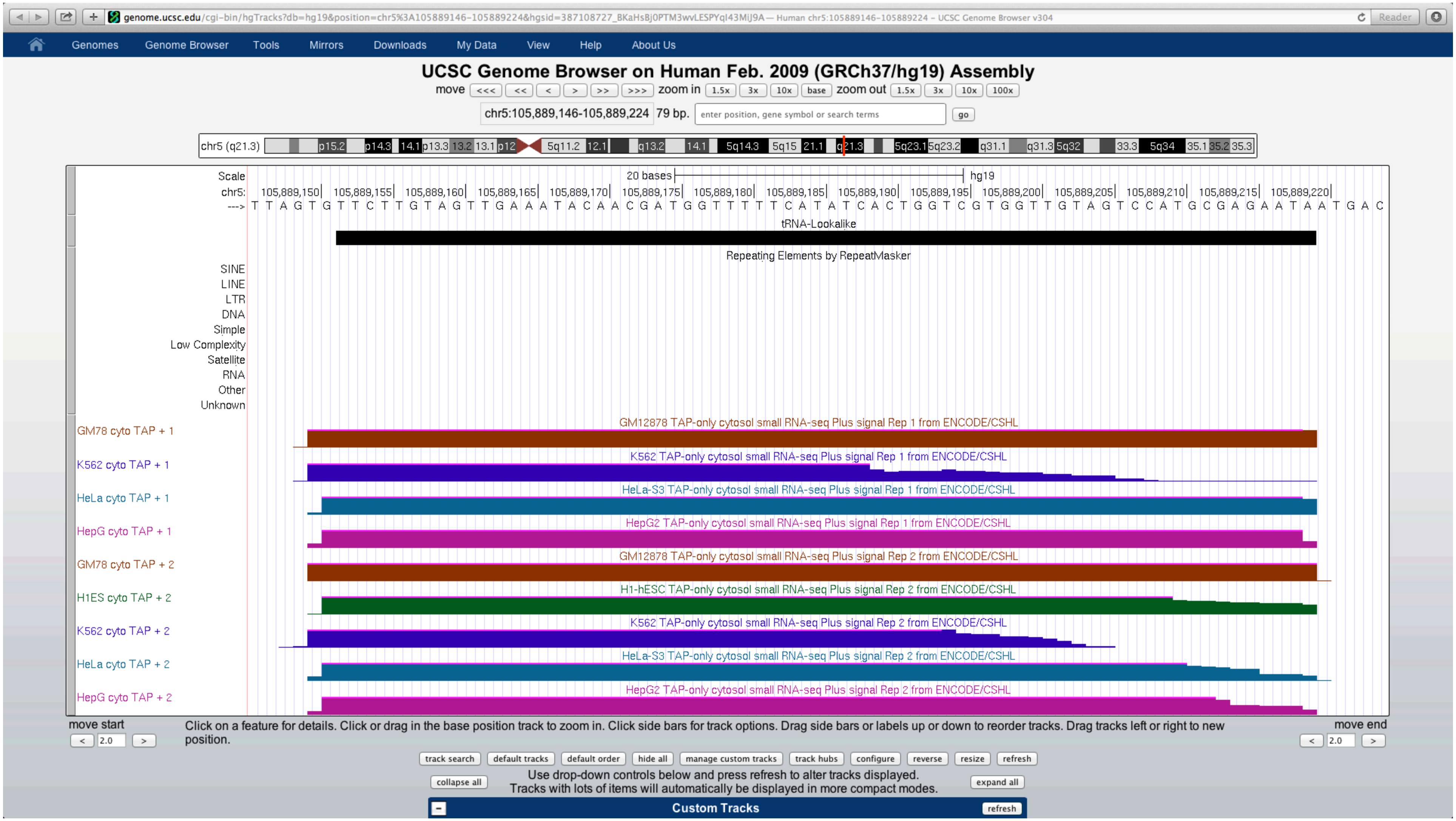

GluTTC chrMT strand (-) 14674-14742 / tRNA-Lookalike at chr5, strand (+) 105889151-105889219

Supp. File S4

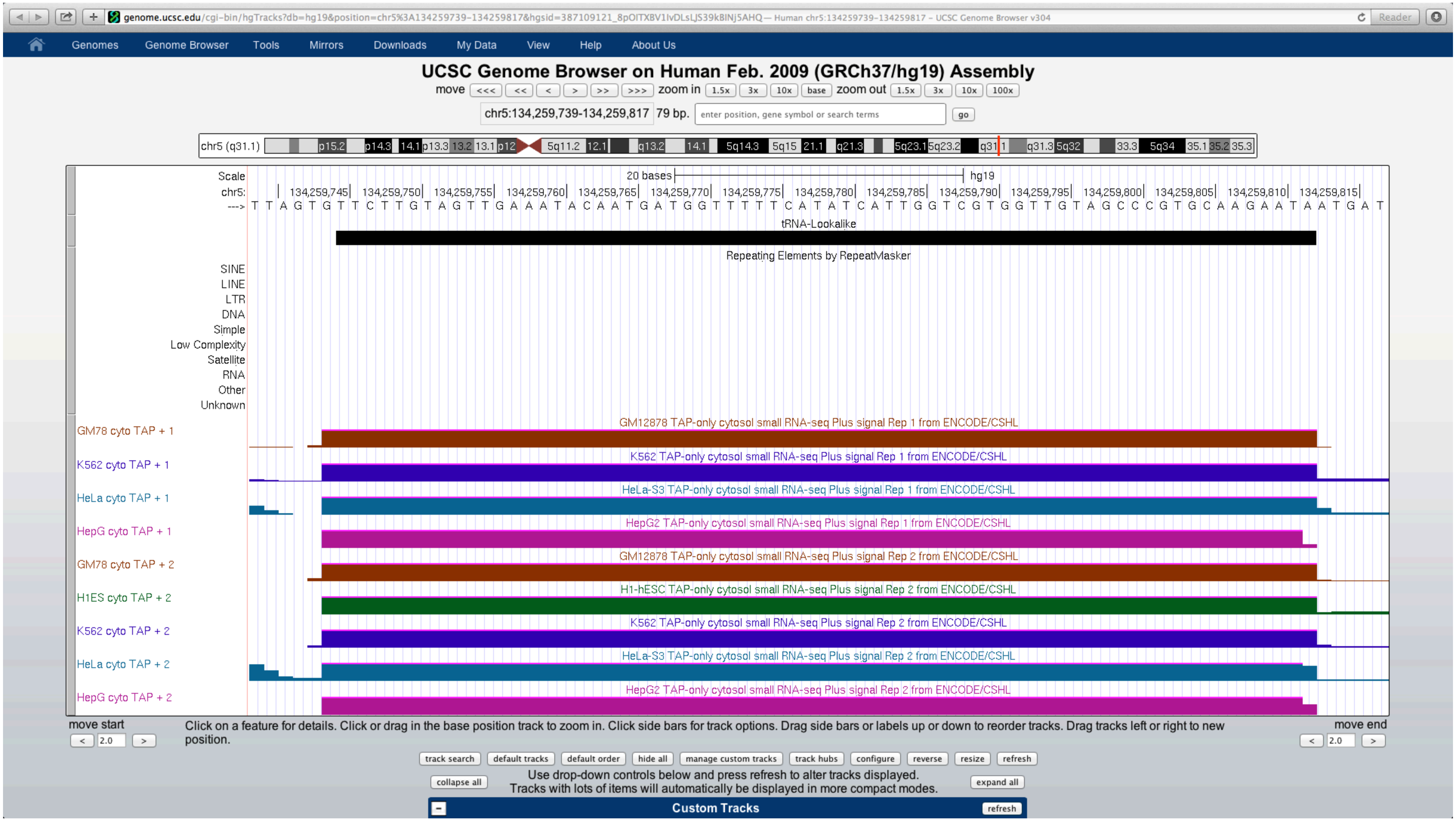

GluTTC chrMT strand (-) 14674-14742 / tRNA-Lookalike at chr5, strand (+) 134259744-134259812

Supp. File S4

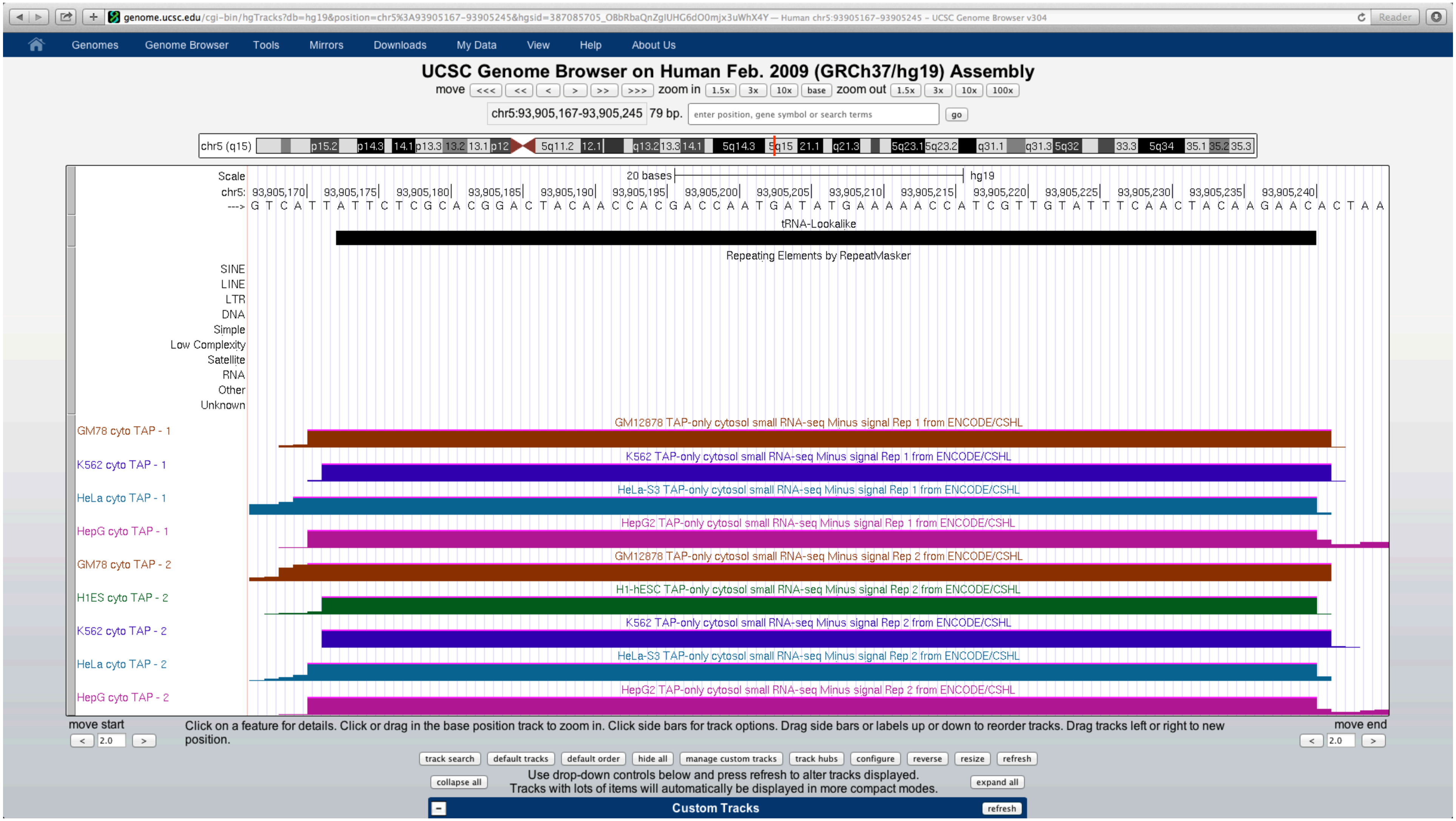

GluTTC chrMT strand (-) 14674-14742 / tRNA-Lookalike at chr5, strand (-) 93905172-93905240

Supp. File S4

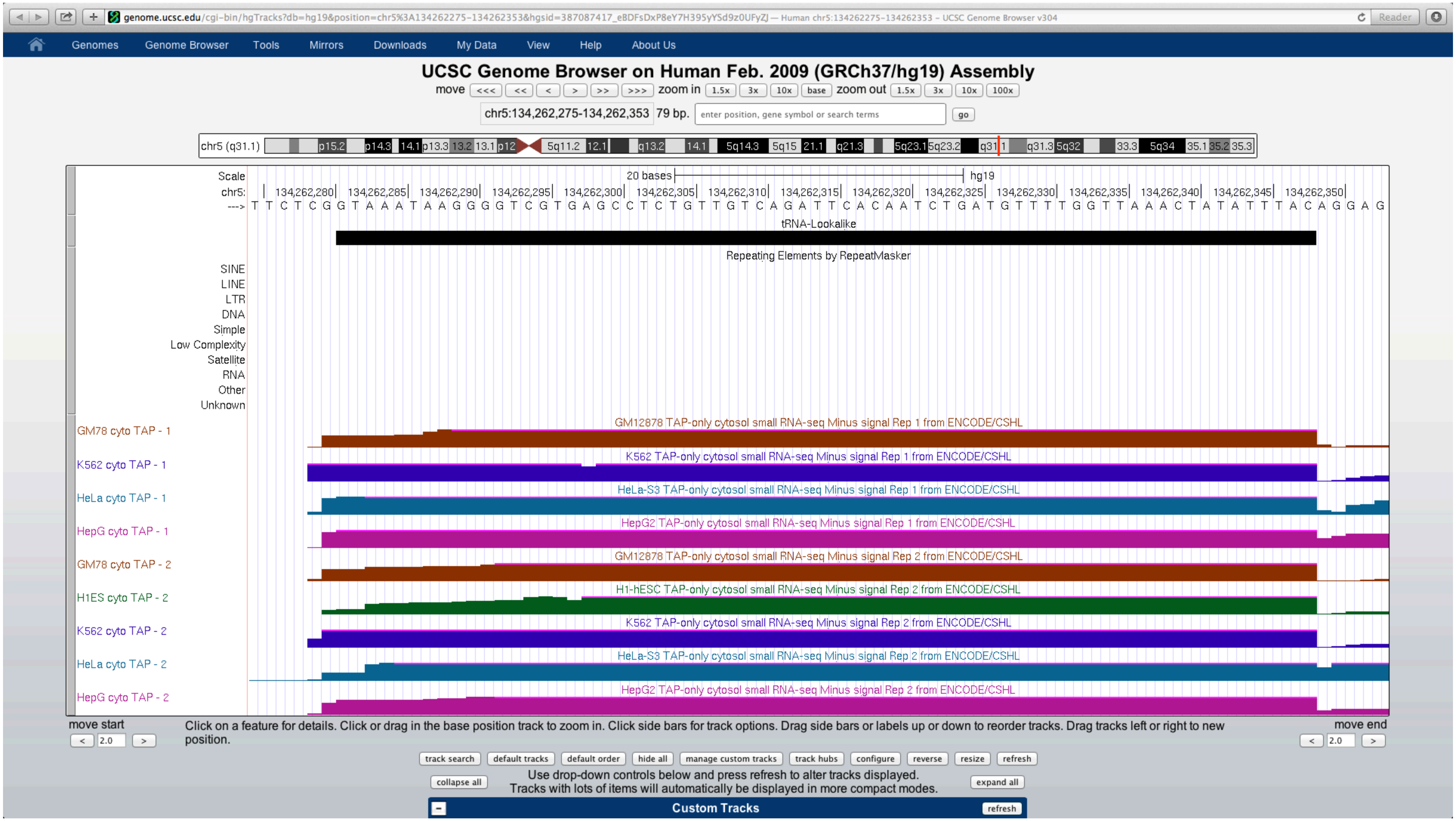

HisGTG chrMT strand (+) 12138-12206 / tRNA-Lookalike at chr5, strand (-) 134262280-134262348

Supp. File S4

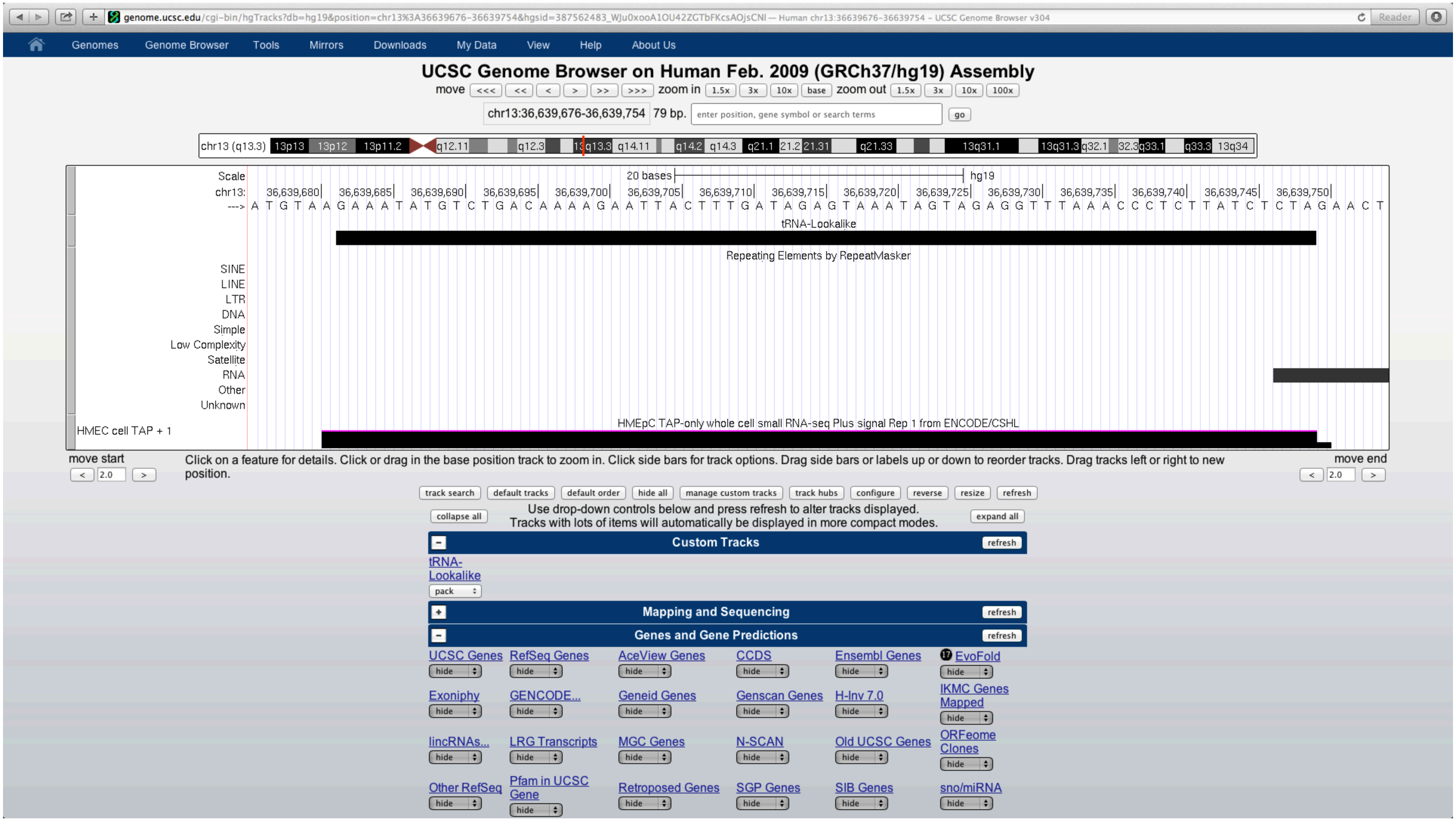

IleGAT chrMT strand (+) 4263-4331 / tRNA-Lookalike at chr13, strand (+) 36639681-36639749

Supp. File S4

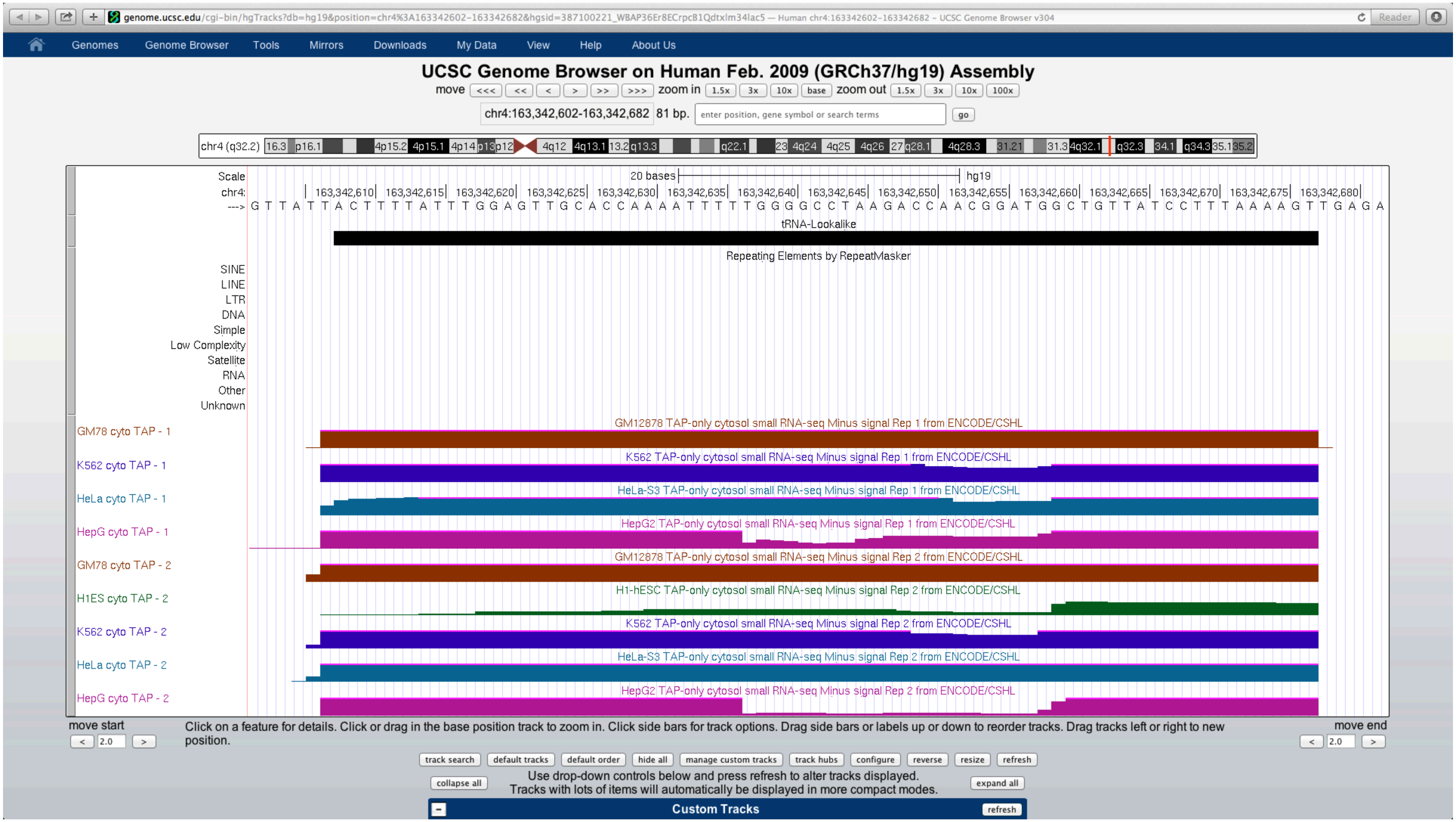

LeuTAG chrMT strand (+) 12266-12336 / tRNA-Lookalike at chr4, strand (-) 163342607-163342677

Supp. File S4

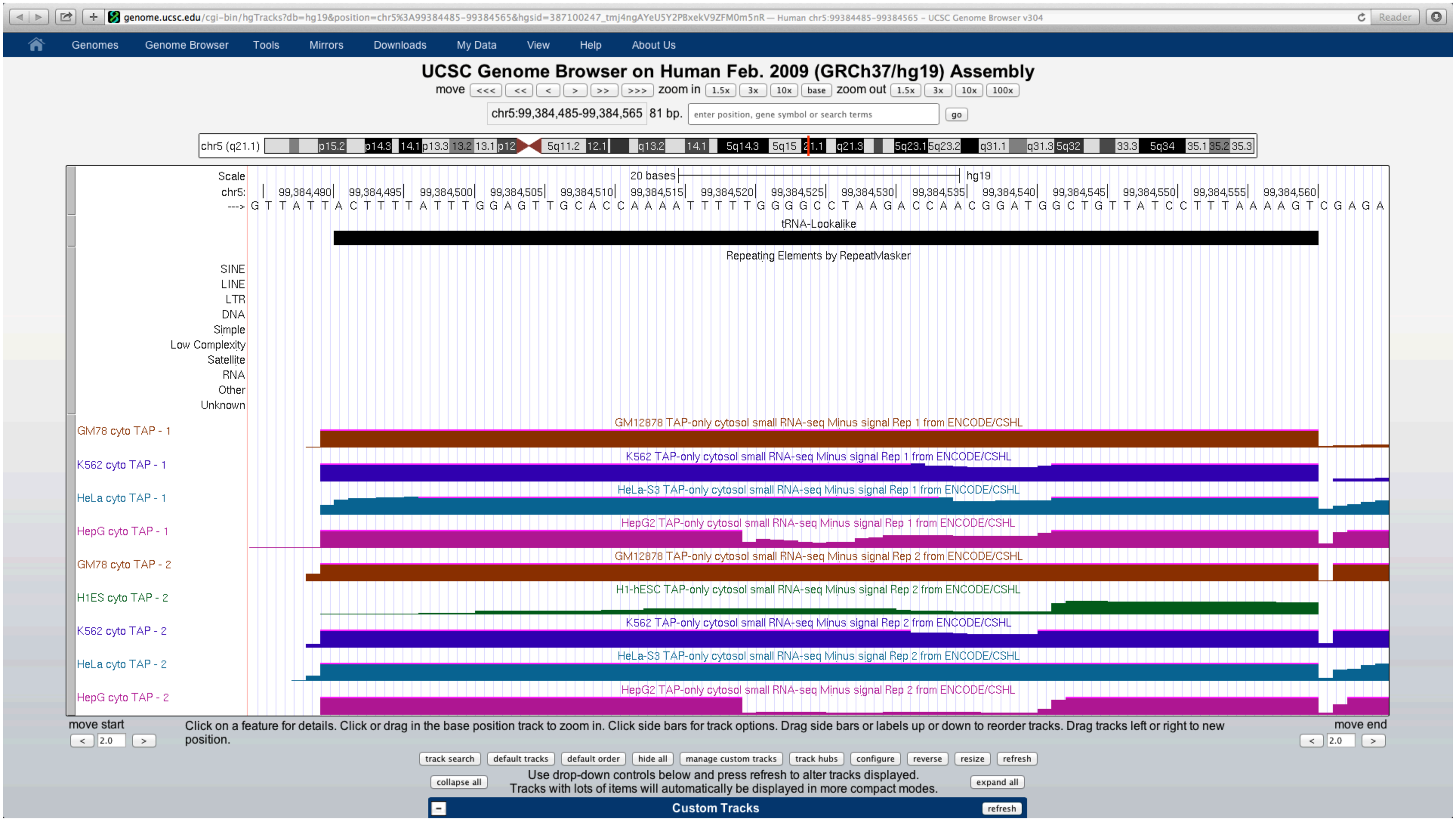

LeuTAG chrMT strand (+) 12266-12336 / tRNA-Lookalike at chr5, strand (-) 99384490-99384560

Supp. File S4

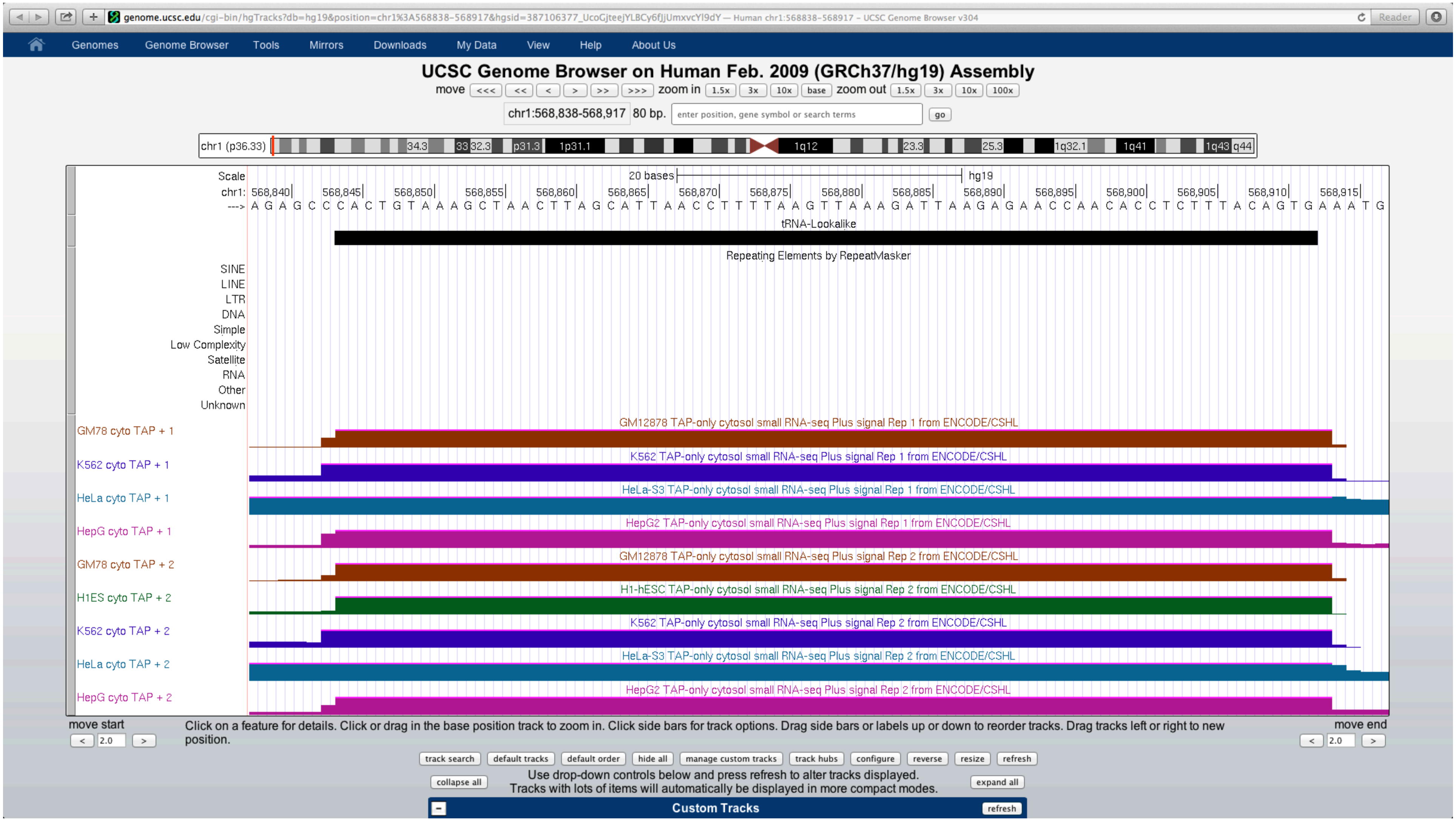

LysTTT chrMT strand (+) 8294-8363 / tRNA-Lookalike at chr1, strand (+) 568843-568912

Supp. File S4

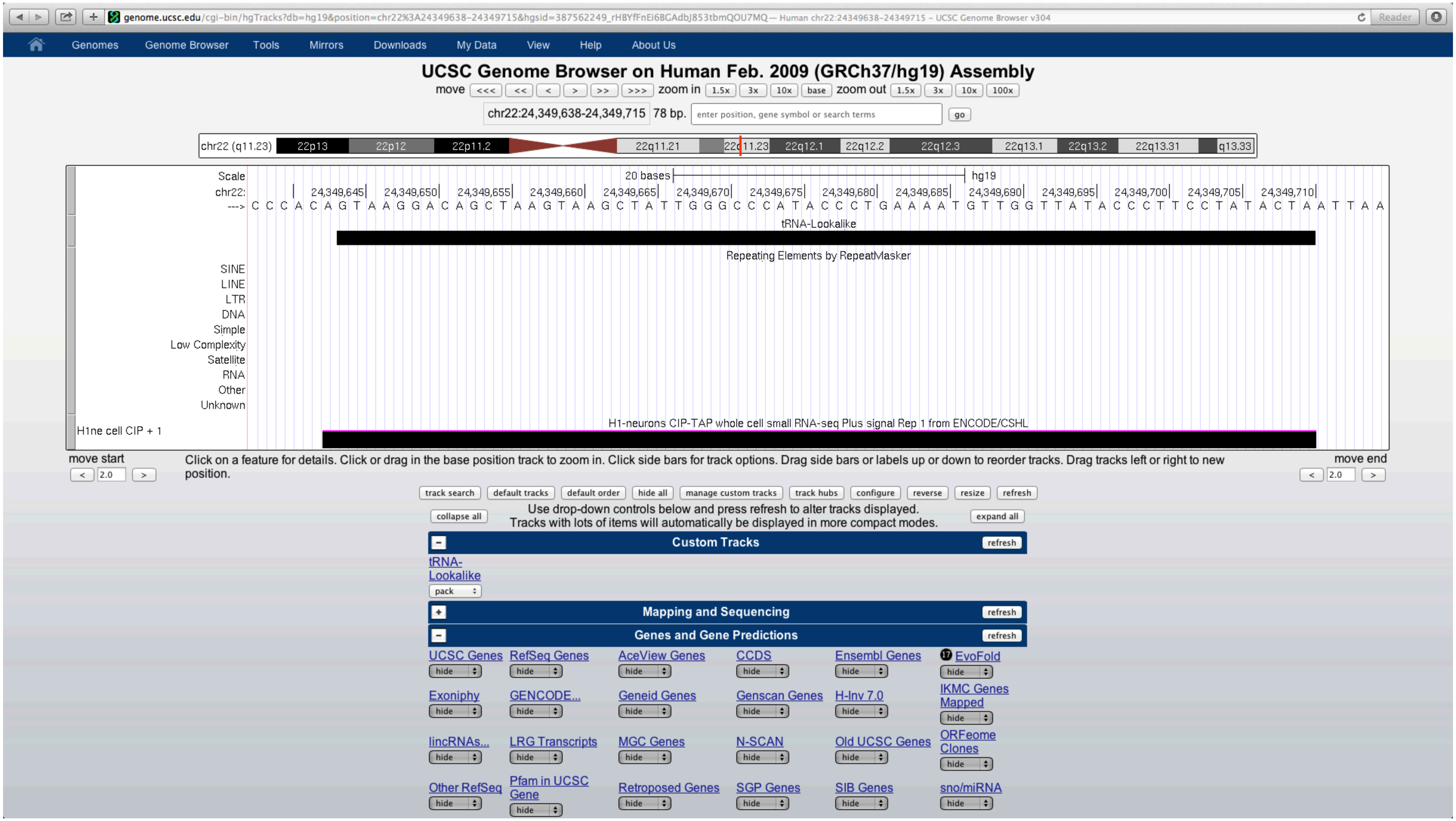

MetCAT chrMT strand (+) 4402-4469 / tRNA-Lookalike at chr22, strand (+) 24349643-24349710

Supp. File S4

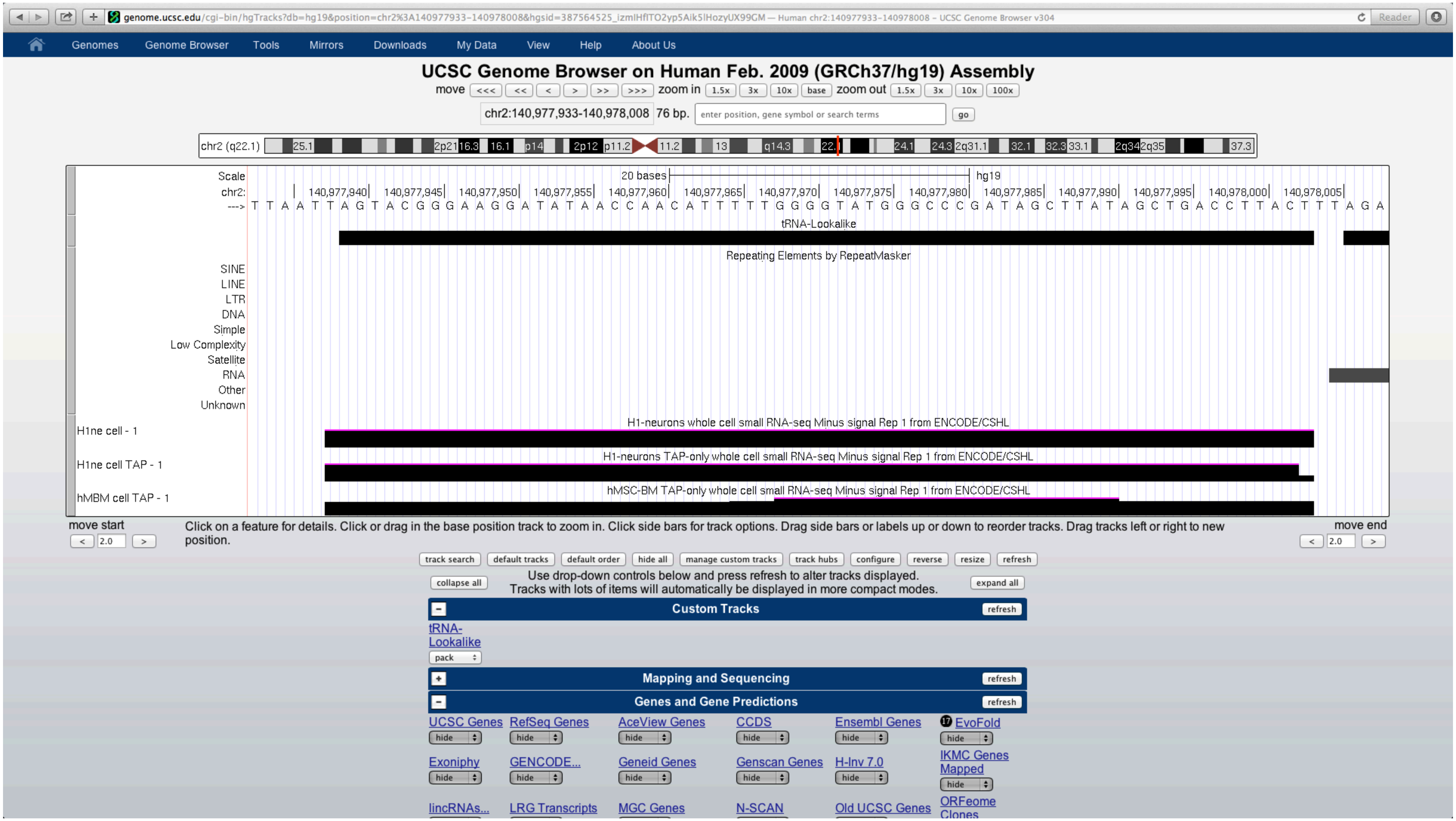

MetCAT chrMT strand (+) 4402-4469 / tRNA-Lookalike at chr2, strand (-) 140977938-140978003

Supp. File S4

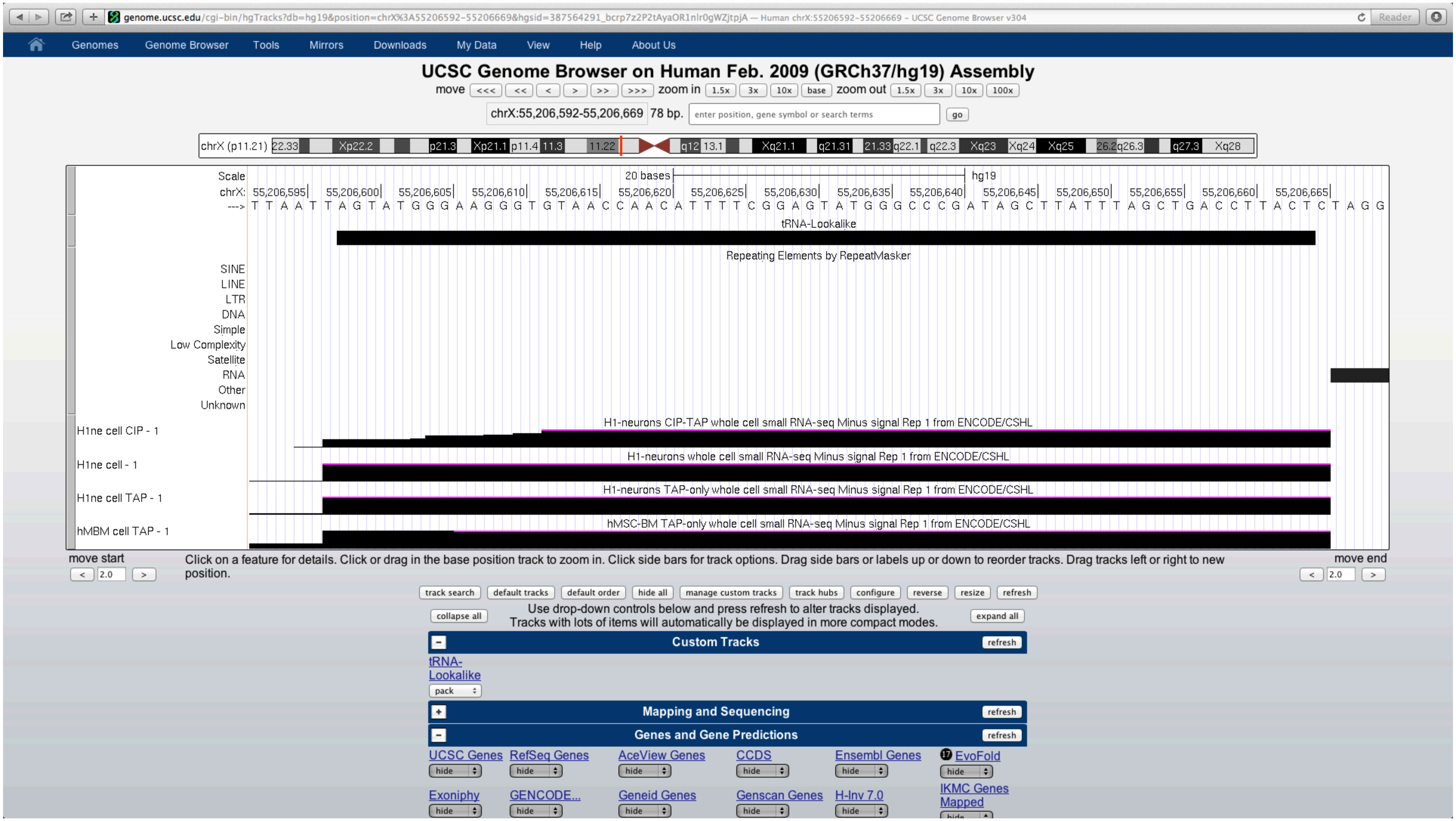

MetCAT chrMT strand (+) 4402-4469 / tRNA-Lookalike at chrX, strand (-) 55206597-55206664

Supp. File S4

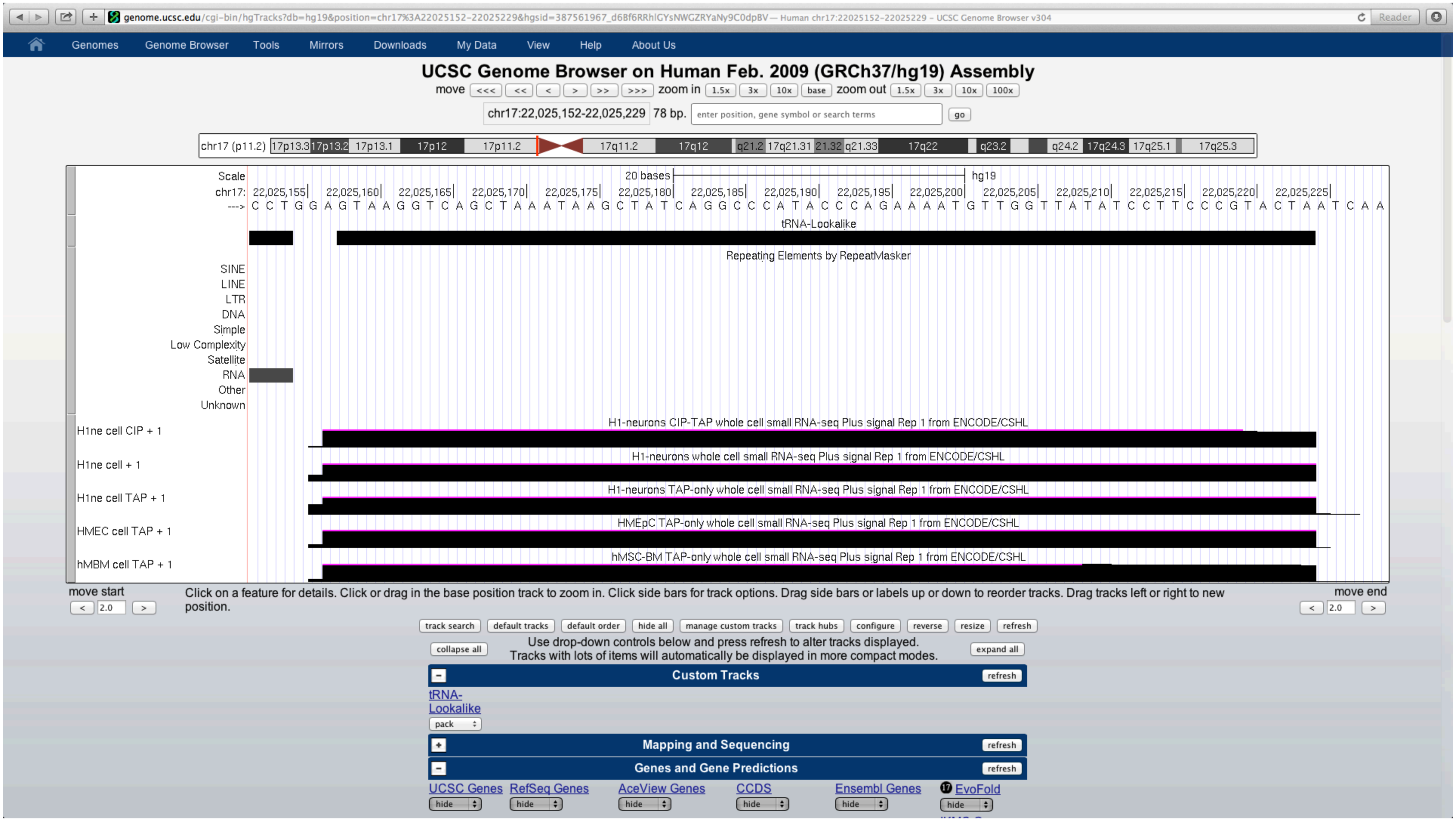

MetCAT chrMT strand (+) 4402-4469 / tRNA-Lookalike at chr17, strand (+) 22025157-22025224

Supp. File S4

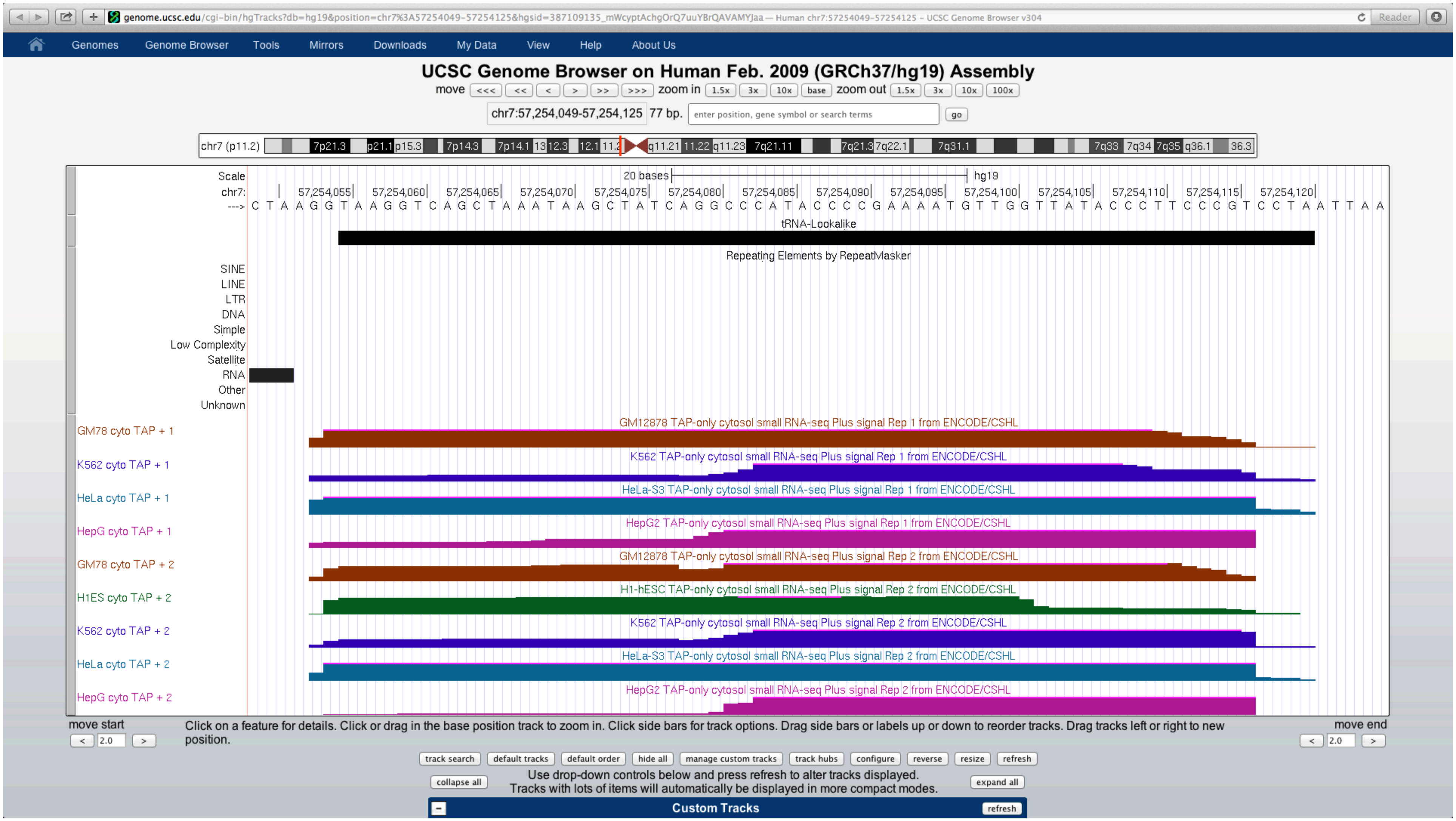

MetCAT chrMT strand (+) 4402-4469 / tRNA-Lookalike at chr7, strand (+) 57254054-57254120

Supp. File S4

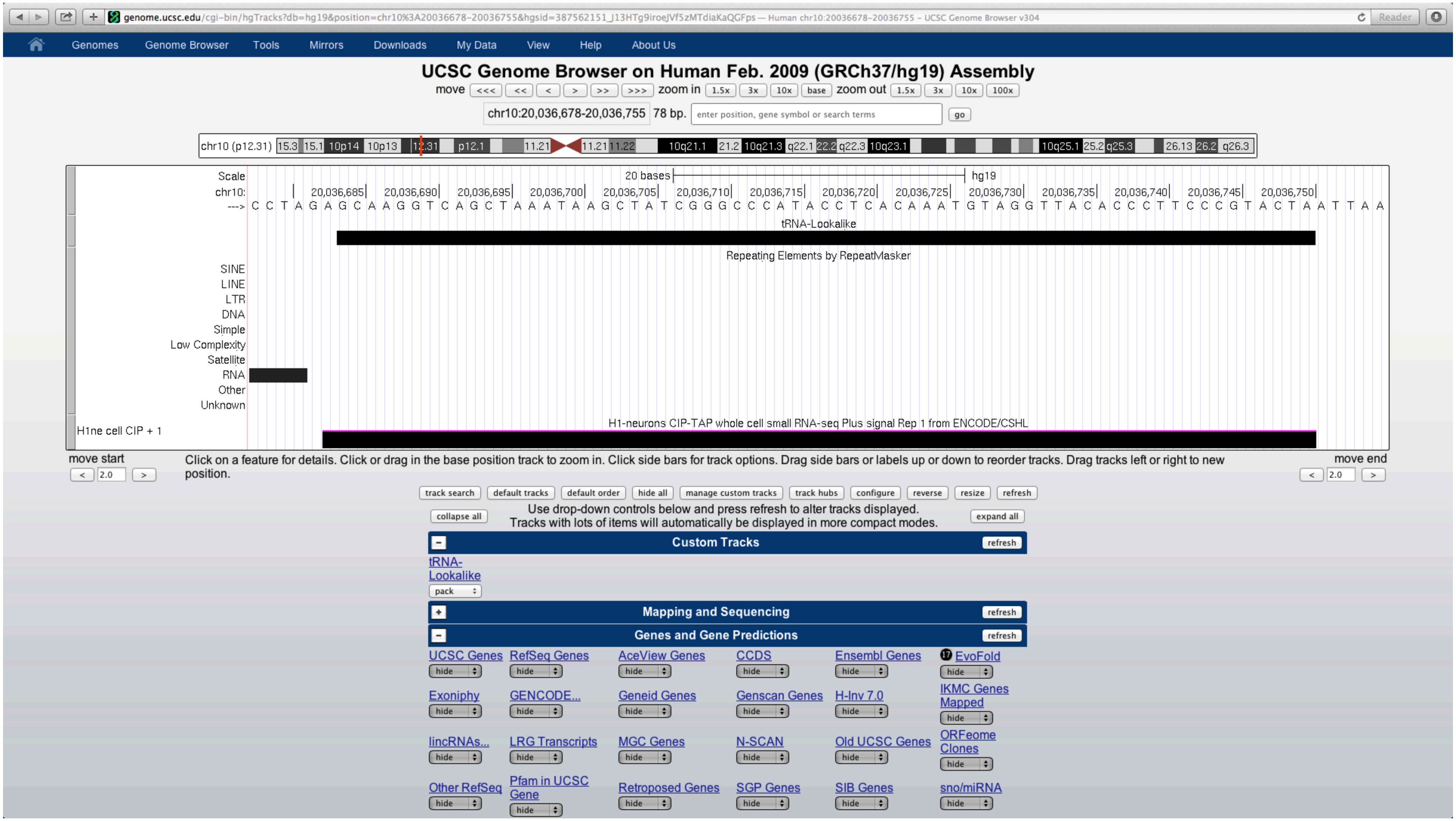

MetCAT chrMT strand (+) 4402-4469 / tRNA-Lookalike at chr10, strand (+) 20036683-20036750

Supp. File S4

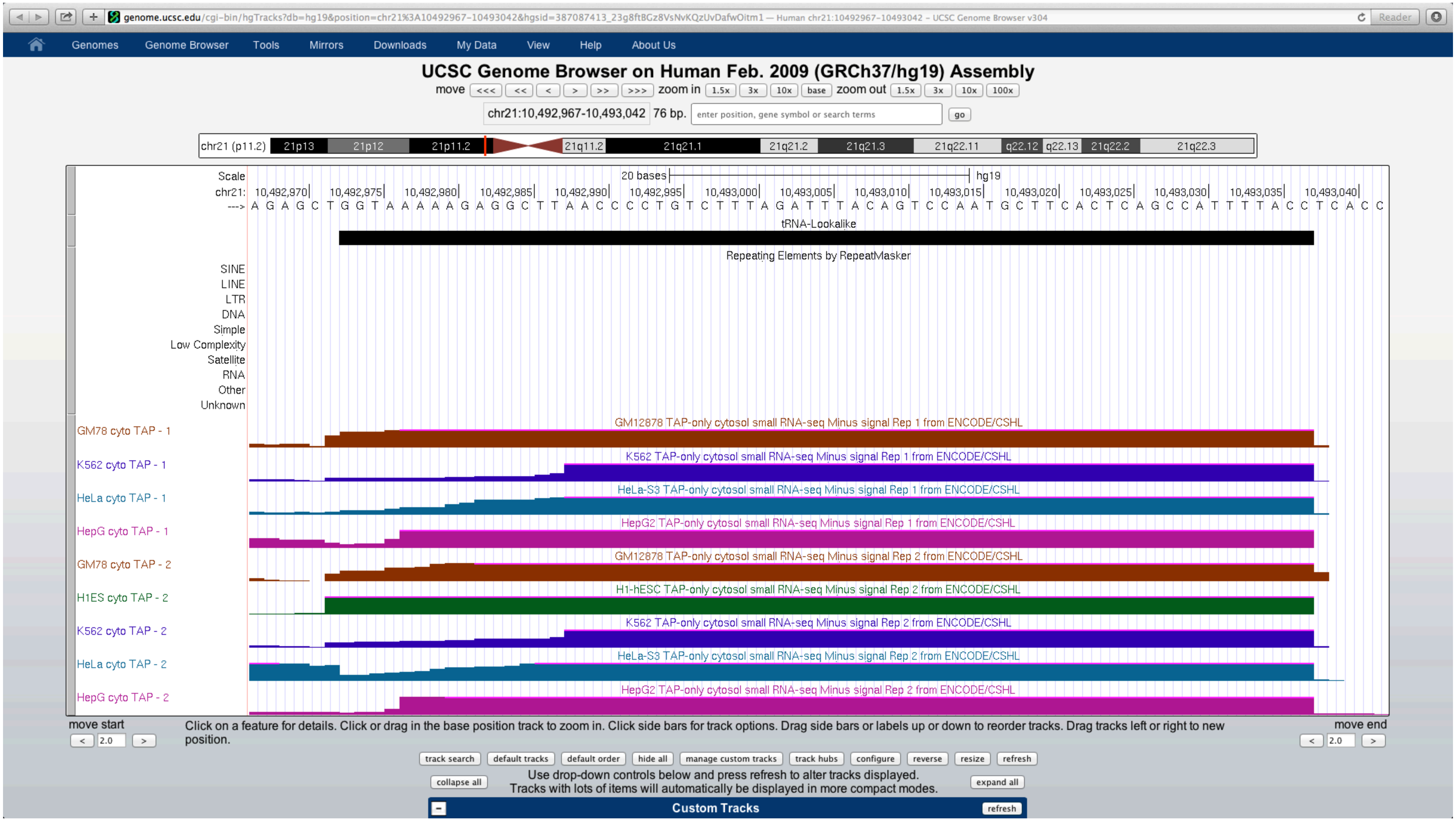

TyrGTA chrMT strand (-) 5826-5891 / tRNA-Lookalike at chr21, strand (-) 10492972-10493037

Supp. File S4

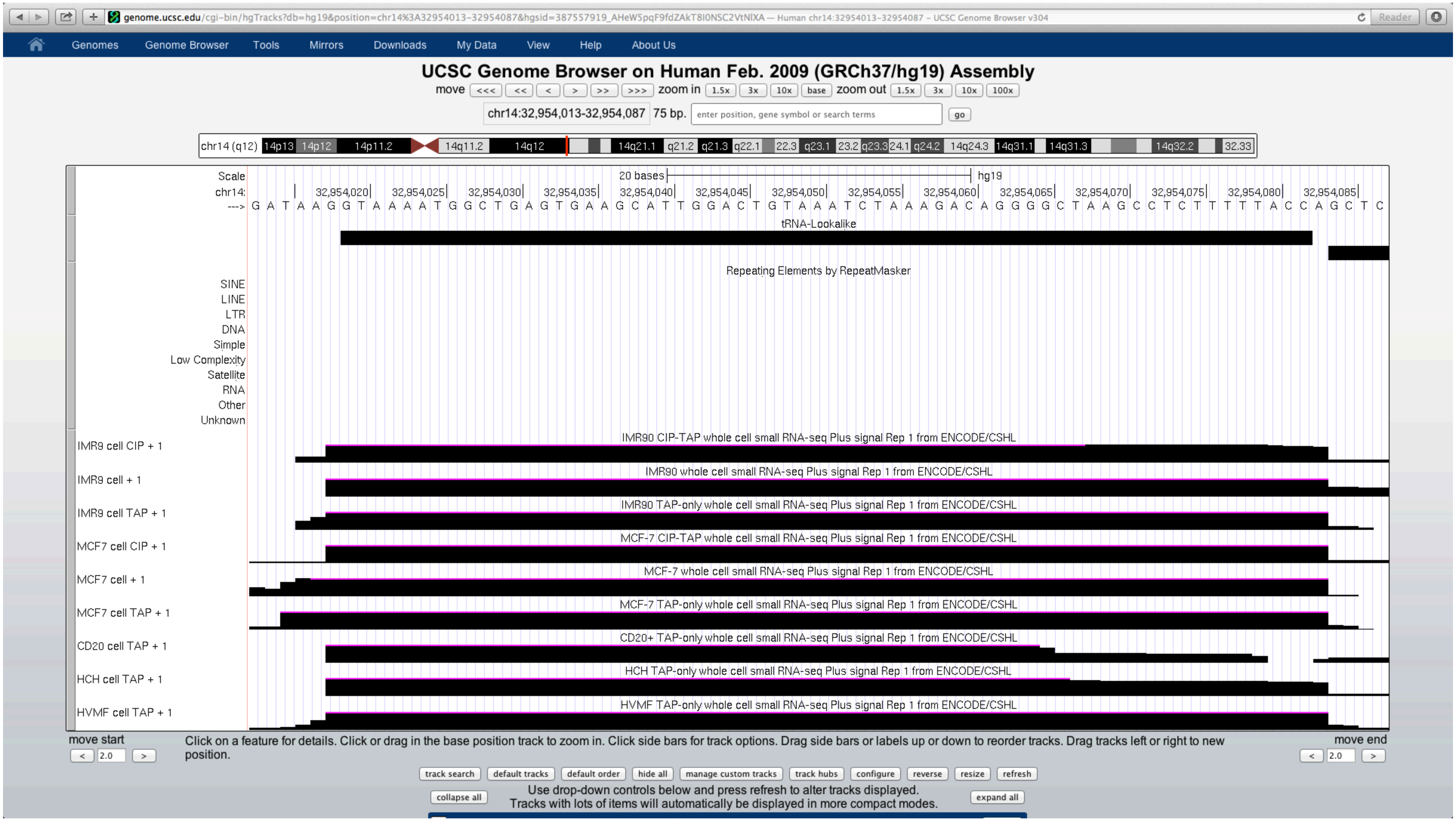

TyrGTA chrMT strand (-) 5826-5891 / tRNA-Lookalike at chr14, strand (+) 32954018-32954082

Supp. File S4

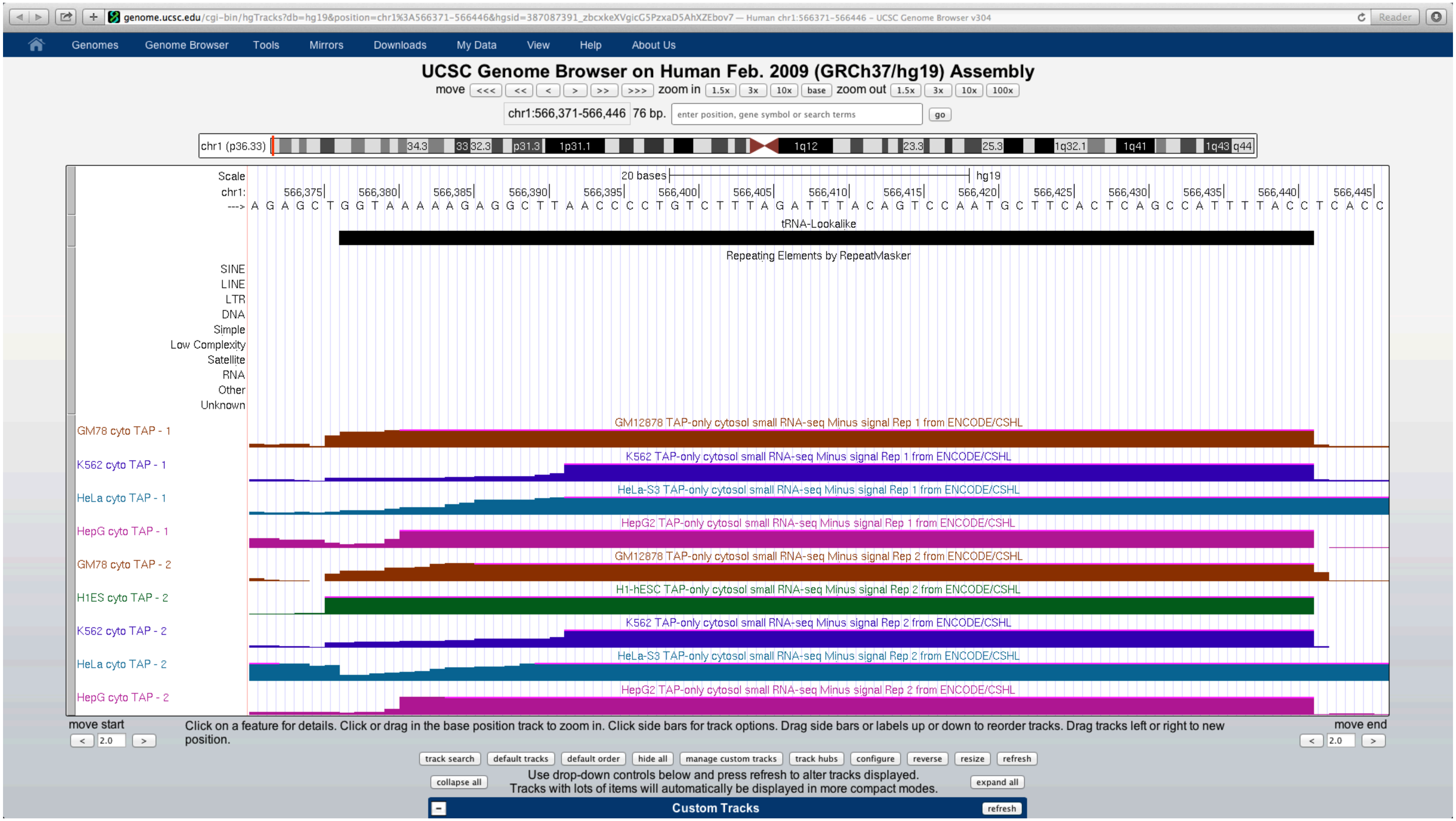

TyrGTA chrMT strand (-) 5826-5891 / tRNA-Lookalike at chr1, strand (-) 566376-566441
