# Supplementary material for: Nuclear and mitochondrial tRNA-lookalikes in the human genome
Source: Front Genet. 2014 Oct 8;5:344. doi: 10.3389/fgene.2014.00344 (PMC4189335; doi:10.3389/fgene.2014.00344)
Supplement: Supplementary file 5 [file DataSheet5.PDF]

Supp. File S5

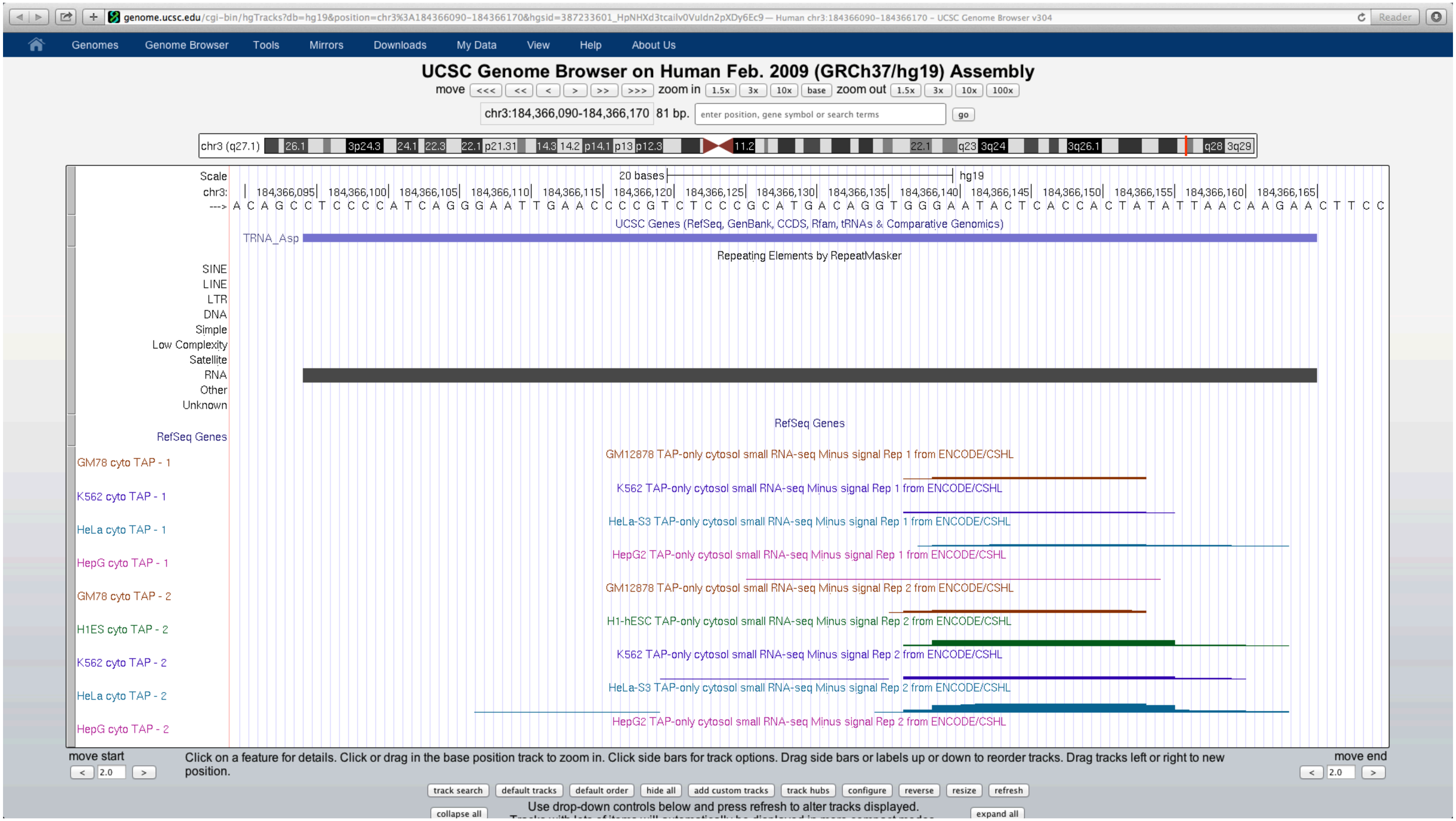

trna3 AspGTC at chr3 strand (-) 184366095-184366165

Supp. File S5

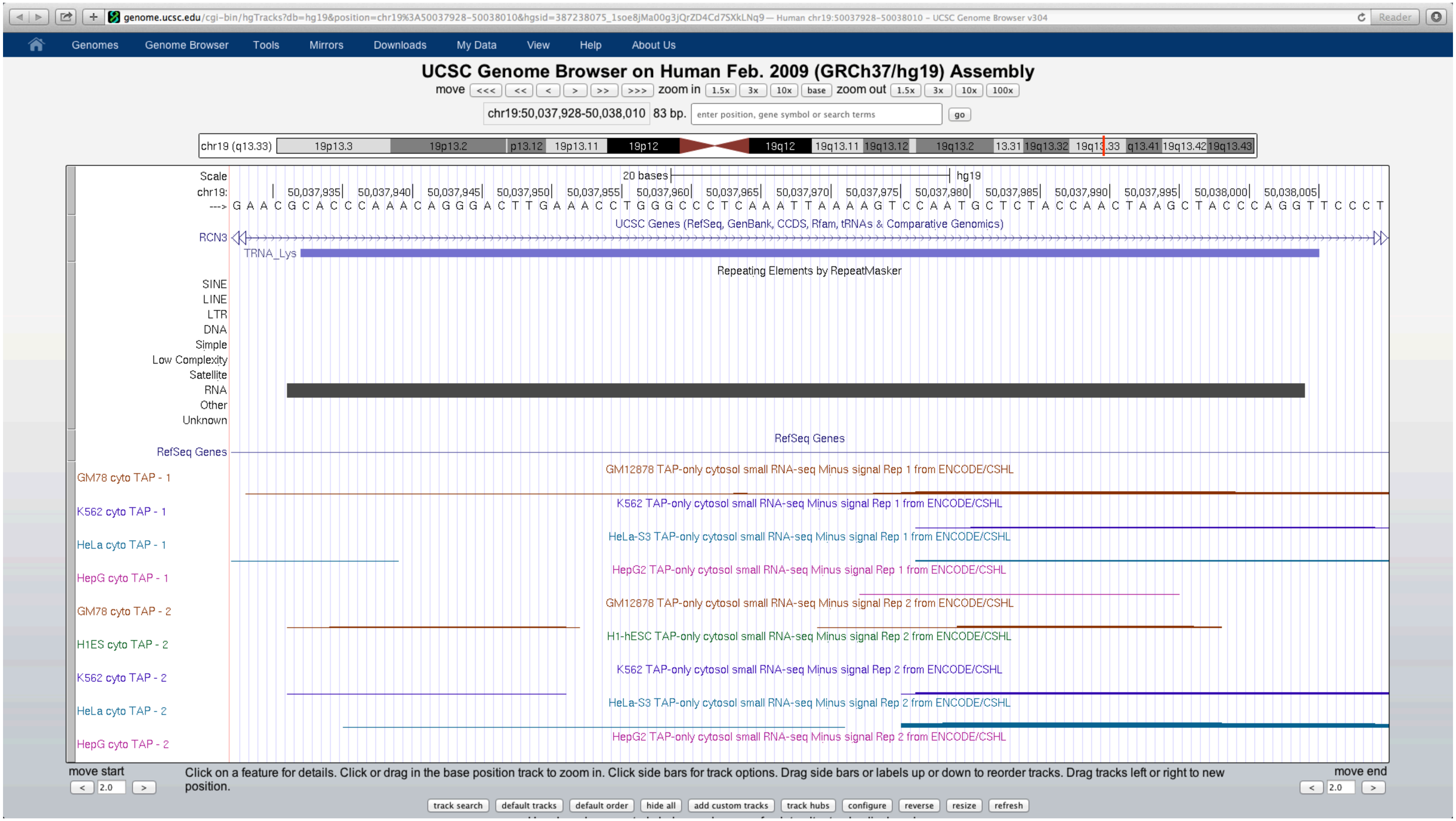

trna7 LysTTT at chr19 strand (-) 50037933-50038005

Supp. File S5

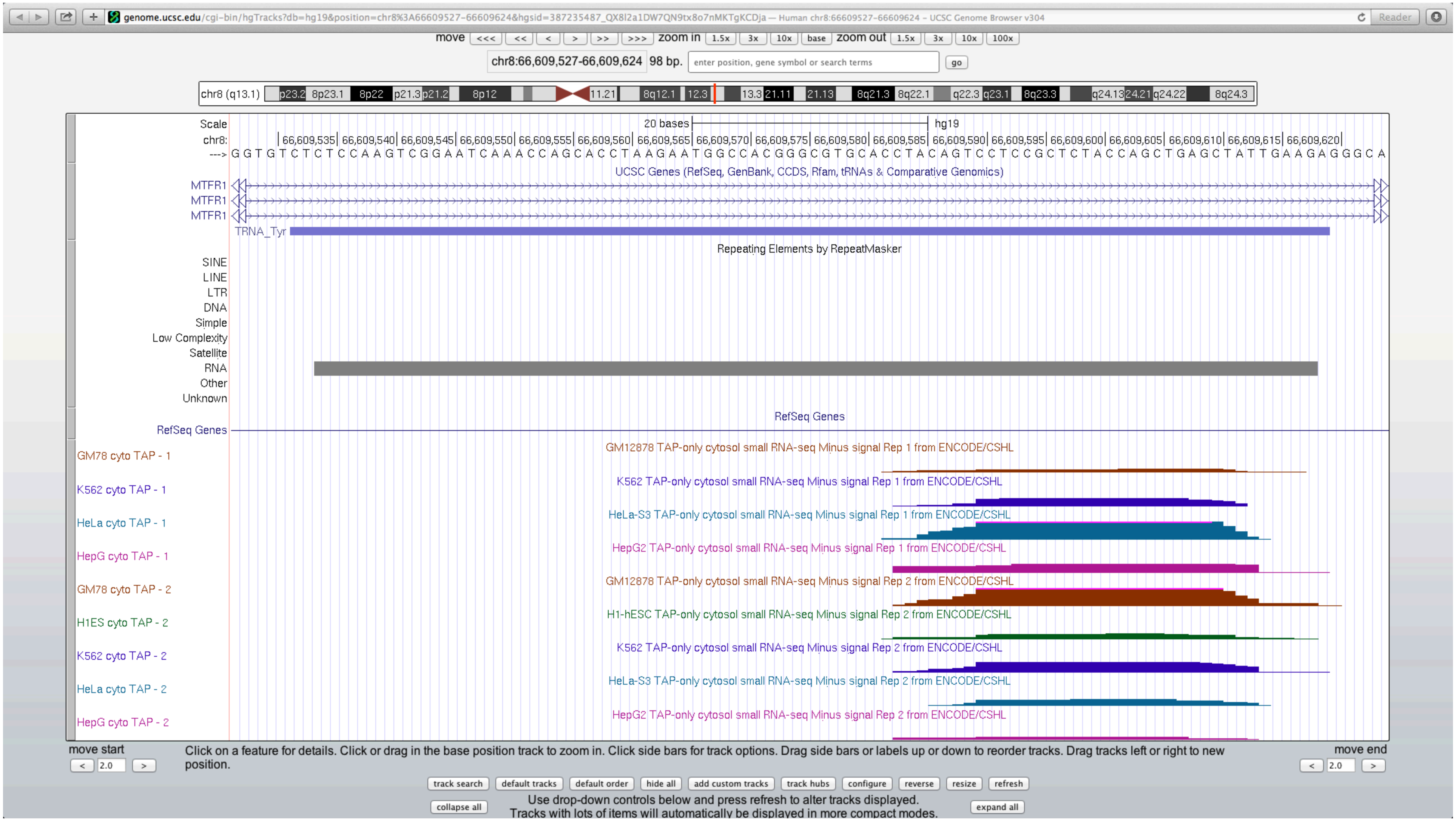

trna12 TyrGTA at chr8 strand (-) 66609532-66609619

Supp. File S5

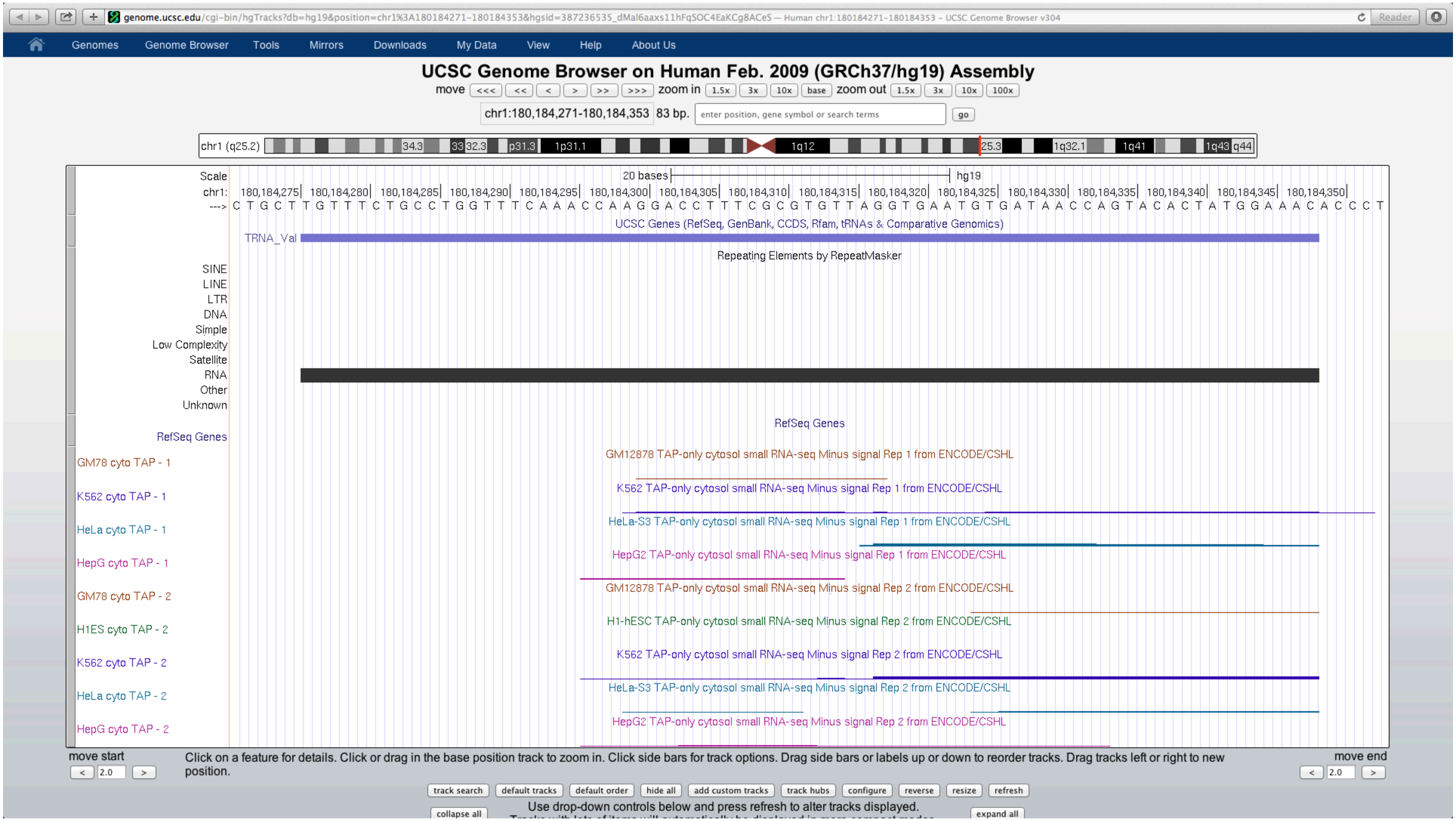

trna12 TyrGTA at chr8 strand (-) 66609532-66609619
