# Supplementary material for: Nuclear and mitochondrial tRNA-lookalikes in the human genome
Source: Front Genet. 2014 Oct 8;5:344. doi: 10.3389/fgene.2014.00344 (PMC4189335; doi:10.3389/fgene.2014.00344)
Supplement: Supplementary file 6 [file DataSheet6.PDF]

# Supp. File S6

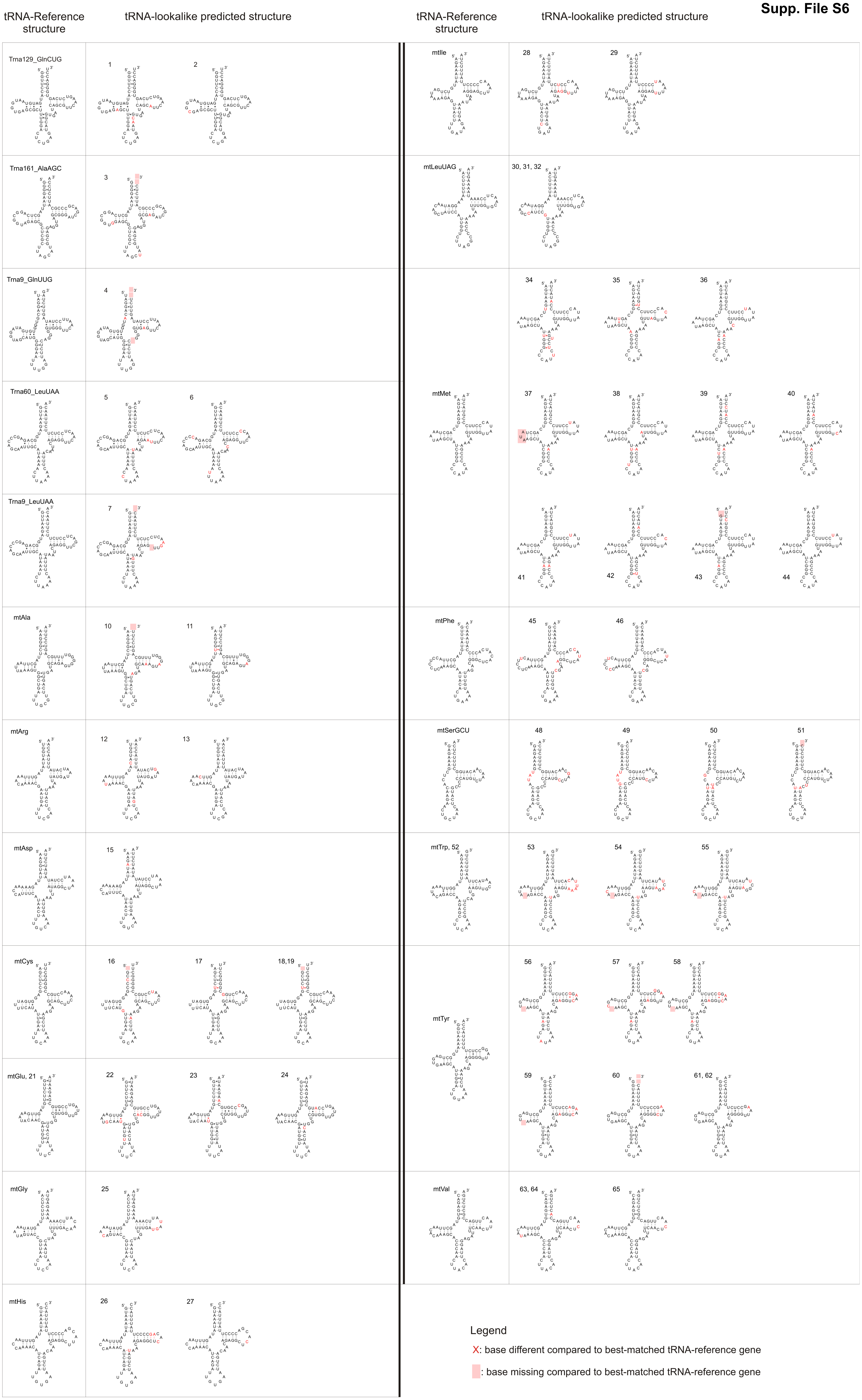


Coordinates of tRNA-lookalikes

1:chr1|+|21740714|21740785|
2:chr1|+|16879957|16880028|
3:chr6|+|26752630|26752701|
4:chr7|+|63571998|63572066|
5:chr10|-|20036367|20036441|
6:chr17|+|22023985|22024059|
7:chr10|-|37890300|37890372|
8:chr14|+|32954252|32954320|
9:chr1|-|566137|566205|
10:chr17|-|22026338|22026405|
11:chr2|+|156120525|156120593|
12:chr5|-|99386357|99386421|
13:chr5|-|134264017|134264081|
14:chr1|-|566207|566279|
15:chr1|+|568069|568136|
16:chr2|-|131031334|131031398|
17:chr14|+|32954083|32954148|
18:chr1|-|566311|566375|
19:chr21|-|10492907|10492971|
20:chr1|-|564879|564950|
21:chr5|-|93905172|93905240|
22:chr5|+|99382083|99382151|
23:chr5|+|134259744|134259812|
24:chr5|+|105889151|105889219|
25:chr5|-|99386768|99386835|
26:chr5|-|99384620|99384688|
27:chr5|-|134262280|134262348|
28:chr12|-|42093125|42093193|
29:chr1|+|564813|564881|
30:chr4|-|163342607|163342677|
31:chr5|-|134262150|134262220|
32:chr5|-|99384490|99384560|
33:chr1|+|568843|568912|
34:chr9|+|5095158|5095226|
35:chr9|-|81357661|81357728|
36:chr1|-|238105793|238105860|
37:chr2|-|140977938|140978003|
38:chr6|-|95156841|95156908|
39:chr7|-|63571929|63571996|
40:chr17|+|19506660|19506727|
41:chr17|+|22025157|22025224|
42:chrX|-|55206597|55206664|
43:chr7|+|57254054|57254120|
44:chr1|+|564952|565019|
45:chr5|-|79947884|79947954|
46:chr11|-|10531757|10531827|
47:chr1|-|567997|568065|
48:chr1|+|181391985|181392043|
49:chr7|+|57261851|57261909|
50:chr5|-|134262221|134262279|
51:chr5|-|99384562|99384619|
52:chr1|+|566062|566129|
53:chr11|-|103276875|103276942|
54:chr8|-|70015240|70015307|
55:chr9|-|95301816|95301883|
56:chr11|+|103276577|103276642|
57:chr2|+|203484634|203484699|
58:chr7|+|141501873|141501938|
59:chr9|-|5096587|5096652|
60:chr14|+|32954018|32954082|
61:chr1|-|566376|566441|
62:chr21|-|10492972|10493037|
63:chr3|-|96337080|96337148|
64:chr5|-|79946867|79946935|
65:chr11|-|10530736|10530804|

The following entries match 100% to the respective tRNA-Referene gene: 8, 9, 14, 20, 21, 33, 47, 52

Supp. File S6
